# Characterization of the neuropathic pain component contributing to myalgia in patients with myotonic dystrophy type 1 and 2

**DOI:** 10.3389/fneur.2024.1414140

**Published:** 2024-08-13

**Authors:** Viviane Schmitt, Petra Baeumler, Anne Schänzer, Dominik Irnich, Benedikt Schoser, Federica Montagnese

**Affiliations:** ^1^Friedrich-Baur-Institut, Department of Neurology, Ludwig Maximilian University (LMU), Munich, Germany; ^2^Multidisciplinary Pain Centre, Department of Anesthesiology, LMU University Hospital, Ludwig Maximilian University, Munich, Germany; ^3^Institute of Neuropathology, Justus Liebig University, Giessen, Germany

**Keywords:** myotonic dystrophy, myalgia, quantitative sensory testing, pain, small fiber neuropathy

## Abstract

**Introduction:**

Chronic muscle pain is common in myotonic dystrophies (DM). Little is known about its pathophysiology. We aimed to investigate the characteristics of the neuropathic pain component contributing contributes to the pathogenesis of chronic pain in DM.

**Methods:**

Twenty-one DM1 and 32 DM2 patients completed pain questionnaires (Brief pain inventory–BPI, PAIN-DETECT, pain disability index–PDI) and underwent neurological examination, nerve conduction studies (NCS), quantitative sensory testing (QST, dorsum of the right hand and right thigh) and skin biopsy to determine the intraepidermal nerve fiber density (IENFD, distal and proximal site of lower extremity). NCS and QST results at the thigh were compared to 27 healthy controls and IENFD and QST at the dorsum of the hand to published reference values.

**Results:**

The sensory profile of DM2 patients was characterized by a loss in thermal and mechanical detection, while DM1 patients showed reduced mechanical and heat pain thresholds and higher mechanical pain sensitivity. Both DM groups showed pressure hyperalgesia. IENFD was reduced in 63% of DM1 patients and 50% of DM2. The slightly higher pain interference and disability found in DM2 was rather due to age difference than disease.

**Conclusion:**

Similar pain mechanisms likely occur in both DM1 and DM2, even though a tendency toward more pain sensitivity was observed in DM1 and more sensory loss in DM2. Both QST and reduced IENFD highlight the presence of peripheral nerve damage in DM. This must be considered for the best pain management strategies.

## 1 Introduction

Myotonic dystrophy types 1 and 2 (DM1, DM2) are rare, inherited diseases characterized by heterogeneous multi-systemic involvement (1). The genetic cause of DM1 is a CTG repeat expansion in the 3′-untranslated region (3′UTR) of the DMPK (dystrophia myotonica protein kinase) gene. In contrast DM2 results from a CCTG repeat expansion in intron 1 of the CNBP (cellular nucleic acid-binding protein) gene (2). Both genes are involved in muscle function, and the clinical phenotypes of DM1 and DM2 overlap. More than 60% of patients complain of chronic muscle pain. This is usually described as cramp-like, exercise-related, cold- and palpation-induced pain, involving multiple body regions (spine, proximal and distal muscles), often with a radiating tendency (3–6). In addition to myalgia, other common complaints are arthralgia, headache and abdominal pain. Some studies suggest similarities with the widespread chronic pain seen in fibromyalgia (7, 8). Furthermore, many patients, especially those affected by DM2, consider pain one of the most disabling symptoms and report an unsatisfactory response to common analgesics (5, 9).

The pathophysiology of pain in myotonic dystrophies is not fully understood (3, 8, 10, 11). Pain does not consistently correlate with myotonia, weakness or disease severity. Musculoskeletal impairment due to muscle atrophy, weakness and imbalance with consequent nociceptive pain has been suggested, at least for the commonly reported low back pain (6). Further studies identified an association between pain and psychological factors, such as anxiety and depression (5). Moshurab et al. examined 35 DM2 patients with quantitative sensory testing (QST) and transcriptome analysis on muscle biopsies to better understand the molecular mechanisms of pain in DMs. They found lower pressure pain thresholds (PPT), elevated warm and mechanical detection thresholds (WDT, MDT) as well as elevated mechanical pain thresholds in comparison to healthy controls. Furthermore mechanical pain sensitivity (MPS) and wind-up ratios (WUR) were higher in myalgic patients than in non-myalgic patients (8). In addition, Moshurab et al. found that some genes expressed in muscles were upregulated or downregulated in myalgic vs. non-myalgic patients and hypothesized the presence of peripheral sensitization mechanisms triggering central sensitization. However, some DM2 patients in this study also had diabetes, a known cause of altered QST due to damage of peripheral small nerve fibers. Skin biopsies to detect small fiber reduction were not performed. Thus, reduced intraepidermal nerve fiber density (IENFD) as a possible additional cause for the changes in QST has yet to be investigated. Particularly interesting is that Moshurab et al. found some similarities between the QST profile of DM2 patients and that of fibromyalgia patients. Skin biopsy studies in fibromyalgia patients suggest that reduced IENFD contributes to the pathophysiology of chronic pain in these patients (12, 13).

Therefore, this study aims to explore the characteristics of the neuropathic pain component contributing to the pathogenesis of chronic pain in DM patients. Somatosensory profiles and the IENFD were examined in DM1 and DM2 patients with myalgia and compared to healthy controls to identify potential small nerve fiber involvement in particular.

## 2 Materials and methods

### 2.1 Study participants

We informed patients about the study through the national registry for myotonic dystrophies.^1^ The inclusion criteria for the study were: (1) genetically confirmed diagnosis of DM1 or DM2; (2) age between 18 and 65 years; (3) presence of chronic muscle pain defined as persisting or recurring pain for over 3 months. Exclusion criteria were: previous diagnosis of diabetes mellitus, glucose intolerance and/or previously diagnosed polyneuropathy of any cause. In- and exclusion criteria were preliminarily checked during phone calls with the study candidates and were proven on-site by reviewing the medical records. An age- and sex-matched control group of healthy individuals were recruited for comparison. At the first study visit, patients and controls gave their written informed consent to participate.

During the 1.5-year recruitment period we aimed to include as many patients as possible, but at least 20 participants per group. For a non-parametric group comparison with a group size ratio of 1.5, a sample size of 15 and 23 per group was estimated to be sufficient to detect a standardized mean difference of 1 (assuming an alpha-level of 5% and a power of 80%). Based on the published QST-reference values one standardized mean difference represents a clinically relevant difference for all QST parameters.

### 2.2 Study protocol

The study was conducted in accordance with the declaration of Helsinki and the local ethics committee approved the study protocol (LMU project no. 19/499).

The study design consisted of (1) collection of demographic and disease-related data (diagnosis, body mass index, age at onset, disease duration, present neuromuscular complaints, multisystemic involvement, current pain medication); (2) completion of pain questionnaires [brief pain inventory (BPI), pain-DETECT and pain disability index (PDI)]; (3) neurological examination (including muscle impairment rating scale (MIRS) for DM1 patients); (4) quantitative sensory testing (QST); (5) nerve conduction studies (NCS) and (6) skin biopsies quantifying IENFD.

If patients were taking painkillers, such as non-steroidal anti-inflammatory drugs (NSAIDs) or muscle relaxants (e.g., methocarbamol) on demand, they were asked to pause these medications two days before their study visit. Patients regularly taking pain-modulating drugs (e.g., amitriptyline, duloxetine) were allowed to continue the therapy at the usual dosage.

### 2.3 Pain questionnaires

The pain questionnaires were chosen considering the recommendations of the German Research Network for Neuropathic Pain (Deutscher Forschungsverbund Neuropathischer Schmerz—DFNS) and their previous use in studies investigating pain in myotonic dystrophies.

The BPI assesses pain severity and interference in several activities within the last 24 h. It also depicts most painful body regions, describes the use of pain medications and indicates the percentage of pain relief obtained (14).

The pain-DETECT screening questionnaire estimates the likelihood that patients have a neuropathic pain component. The final score ranges from 0 to 38. If the score is below 13, the neuropathic component is unlikely (<15% likelihood), between 13 and 18 it is uncertain and above 18 it is very likely (>90%) (15).

The PDI measures the pain’s impact on the patient’s ability to participate in seven relevant life activities (e.g. occupation, self-care, recreation) on a scale from 0 to 10. Accordingly, the PDI sum-score ranges from 0 to 70, with higher scores indicating greater pain related disability (16).

### 2.4 Quantitative sensory testing

Quantitative sensory testing (QST) followed the test battery standardized and validated by the DFNS (17). QST is a psychophysical examination used to explore the somatosensory function and the presence of hyperalgesia and/or allodynia. It encompasses 13 sensory parameters, including mechanical and thermal detection and pain thresholds.

All investigators received specific training and certification by the DFNS to perform and interpret QST. The same investigator (VS) performed all QST assessments in recruited patients and healthy controls.

The QST was performed at the dorsum of the right hand and at the right thigh in DM patients and at the right thigh in healthy controls. The hand dorsum was chosen as a pain-free region for which the DFNS provides reference data stratified by age and gender. The thigh region was selected as it represents the most painful region in DM patients. Herein, we report a summarized version of the QST protocol validated by the DFNS.

For thermal testing, we used a thermal sensory analyzer (TSA 2001-II, Medoc Ltd. Advanced Medical Systems, Ramat Yishai, Israel). Cold and warm detection thresholds (CDT and WDT), thermal sensory limen (TSL), cold and heat pain thresholds (CPT, HPT), as well as the number of paradoxical heat sensations (PHS) were assessed. The mechanical detection threshold (MDT) was evaluated with a set of von Frey filaments (OptiHair2, MRC systems GmbH, Heidelberg, Germany). The mechanical pain threshold (MPT), the mechanical pain sensitivity (MPS) and the wind-up ratio (WUR) were determined by using a set of pinprick stimulators (PinPrick Stimulator Set, MRC Systems GmbH, Heidelberg Germany). Furthermore, dynamic mechanical allodynia (DMA) was assessed by stroking with a Q-tip, cotton wool and a paint brush also included in the MRC stimulator set. The vibration detection threshold (VDT) was examined with a Rydel-Seiffer tuning fork (64 Hz, x/8 scale; Arno Barthelmes, Tuttlingen, Germany). The pressure pain threshold was assessed by a pressure algometer with a rubber tip of 1 cm^2^ (FPK20, Wagner Instruments, Greenwich, CT, United States).

### 2.5 Nerve conduction studies

Nerve conduction studies (NCS) were performed to rule out the presence of large fiber polyneuropathy. This examination was done after the QST to avoid any impact caused by the discomfort related to the NCS. The following nerves were examined in all patients (DM1, DM2) and controls on the right side: ulnar motor nerve, peroneal motor nerve, sensory radial nerve and sural nerve. For the classification of abnormal NCS, the reference values of our neurophysiology laboratory were adopted.

### 2.6 Intraepidermal nerve fiber density evaluated by skin biopsies

To assess the intraepidermal nerve fiber density (IENFD), two 3 mm diameter skin punch biopsies were taken 10 cm above the lateral malleolus (distal biopsy) and 20 cm below the iliac spine (proximal biopsy). The skin samples were fixed with Zamboni fixative, washed in PBS, transferred to 10% sucrose and stored at −80°C freezer until use. From each biopsy, 50 μm thick frozen sections were stained using a free-floating protocol with primary antibody anti-protein gene product (PGP 9.5, 1:1,000, Zytomed) and secondary antibody goat anti-rabbit Alexa Fluor 488 (1:1,000, Thermo Fisher Scientific) (18). Four sections were mounted with DAPI Fluorshield (Abcam) and were examined using an Olympus IX83 inverted microscope equipped with a UPLSAPO400XO/1.4 objective and a DP 74 digital camera (Olympus, Tokyo, Japan). Image analysis was performed using cell Sens Dimension software (Olympus). During the morphologic analysis, the investigator (FM) was blinded to the patient’s diagnosis (DM1 or DM2). The IENFD was quantified using standardized guidelines and age- and sex-adjusted normative values (19). The proximal/distal IENFD ratio was calculated to evaluate the pattern of small fibers reduction. A ratio < 1 was considered a proximal reduction, > 2.5 a distal reduction.

### 2.7 Statistical analysis

The data were analyzed with SPSS Statistics (Version 27.0, IBM, Armonk, NY) and R (version 4.2.3, R Core Team, 2022). The normality of variables was assessed by the Shapiro–Wilk test. As most continuous variables were non-normally distributed, descriptive statistics are displayed as medians and interquartile ranges (IQR). Categorical variables are reported as absolute and relative frequencies. Comparisons of continuous and ordinal variables between the three study groups were performed by applying the Kruskal-Wallis-test (KW-test) with Dunn’s post-hoc test adopting Bonferroni adjustment for pairwise comparisons. Group comparisons of data that were only collected in DM1 and DM2 patients were performed by the Mann–Whitney U test. Comparisons of nominal and dichotomous data were performed by Chi2 or Fisher-test, respectively. Correlations between outcome measures were evaluated by Spearman correlation. *p*-values < 0.05 were considered significant.

According to DFNS reference data in healthy volunteers, all QST parameters are either normally distributed (CPT, HPT, VDT) or normally distributed in log-space (CDT, WDT, TSL, MDT, MPT, MPS, WUR, PPT, PHS, DMA). This was indeed the case for our healthy control group. However, QST parameters in DM1 and DM2 patients showed skewed distributions. Therefore, the sensory profiles of DM1 and DM2 patients are illustrated as boxplots of the patients’ z-scores, and non-parametric tests as stated above were used for inferential statistics.

Z-Scores were calculated by the following formula:


Z=(value patient−mean reference)/SDcomparator

Z-scores below zero indicate a loss of function; z-scores above zero indicate a gain of function. Z-scores for QST-data at the thighs were calculated based on data established in the age- and gender-matched healthy control group. Z-Scores for QST-data at the dorsum of the hand were calculated compared to DFNS reference data as described by Magerl et al. (20). Magerl et al. suggest performing a statistical comparison with the DFNS reference data by computing the t-test statistic from the z-scores of the study sample and an ideal normal distribution with mean 0 and SD 1. Given the skewed distribution in our patient samples, we applied a corresponding non-parametric test strategy. 100 random samples from a normal distribution with mean 0 and SD 1 were compared to z-scores of our samples by applying the KW- and Dunn’s post-hoc tests. The 100th root of the product of the 100 *p*-values is reported as an approximation for the p-value of a comparison with an ideal normal distribution.

Sensitivity analyses for all outcome comparisons with adjustment for either age and gender or BMI and gender were carried out by applying generalized linear models with either an identity (continuous outcomes), a logit (dichotomous outcomes), or a cumlogit (categorical outcomes with more than two categories) link function. Age and BMI were not included in the same model, as this would have caused collinearity (Spearman correlation coefficient (rS) age ~ BMI 0.355 *p* < 0.001).

## 3 Results

### 3.1 Demographic and clinical features

We included 21 DM1 patients, 32 DM2 patients and 27 controls. All subjects were German citizens with the only exception of one participant from Switzerland. All were of Caucasian ethnicity. A significant age difference was detected between the three groups (KW-test *p* = 0.002). This was attributed to an older age of the DM2 cohort than the DM1 cohort (median [IQR] 55.0 [51.3; 57.0] vs. 42.0 [29.5; 52.0], post-hoc p = 0.002). The age of controls (52.0 [34.0; 59.0]) was not different to the age of DM1 (post-hoc *p* = 0.199) and DM2 patients (post-hoc *p* = 0.276). Two thirds in all three groups were female (DM1 13 (62%), DM2 19 (59%), controls 18 (67%), Chi2-test *p* = 0.845). BMI distributions were similar in the three groups with a trend toward an age-related higher BMI among DM2 patients (DM1 23.3 [18.6; 28.7], DM2 26.3 [22.6; 29.6], controls 22.6 [21.6; 25.5], *p* = 0.052). Table 1 summarizes further demographic and clinical characteristics of the two DM patient cohorts.

**Table 1:** Demographic and clinical patient characteristics.

Variable	DM1	DM2	*p*-value
	*n* = 21	*n* = 32	(DM1 vs. DM2)
Age at diagnosis, years (median [IQR])	29.0 [24.0–42.0]	49.0 [41.0–53.0]	**<0.001**
Time since onset, years (median [IQR])	11.0 [7.0; 19.5]	14.5 [6.3; 25.0]	0.434
Marital status [*n* (%)]	7 (33%)	1 (3%)	**0.011**
SINGLE	12 (57%)	26 (81%)	
married	2 (10%)	5 (16%)	
Divorced			
Years of education (median [IQR]) (range)	13,0 [12,5; 17,0] (8–20)	14,0 [12,0; 16,0] (9–20)	0.804
Present job	7 (33%)	13 (41%)	0.232
Full time	4 (19%)	5 (16%)	
Part time	0 (0%)	3 (9%)	
Housework	6 (29%)	9 (28%)	
Retired	1 (5%)	2 (6%)	
Not employed	3 (14%)	0 (0%)	
Other			
**Present neuromuscular complains [*n* (%)]**			
Weakness	19 (90%)	27 (84%)	0.69
Myotonia	17 (81%)	23 (72%)	0.529
Myalgia	20 (95%)	30 (94%)	1
Difficulties swallowing	9 (43%)	8 (25%)	0.232
Difficulties speaking	6 (29%)	3 (9%)	0.131
Other neuromuscular symptoms	4 (19%)	4 (13%)	0.698
**Multisystem involvement [*n* (%)]**			
Daytime sleepiness	18 (86%)	18 (56%)	**0.035**
Hypertension	2 (10%)	18 (56%)	**<0.001**
Hyperlipidemia	3 (14) %	17 (53%)	**0.008**
Cataract	6 (29%)	13 (41%)	0.4
Thyroid disease	5 (24%)	12 (38%)	0.383
Arrhythmia	4 (19%)	10 (31%)	0.362
Depression	2 (10%)	8 (25%)	0.282
Shortness of breath	3 (14%)	4 (13%)	1
Psoriasis	4 (19%)	3 (9%)	0.415
Gall-bladder disorders	2 (10%)	2 (6%)	1
Stroke	3 (14%)	1 (3%)	0.289
Tumor	0 (0%)	3 (9%)	0.269
Rheumatoid arthritis	0 (0%)	1 (3%)	1
Restless legs syndrome	0 (0%)	2 (6%)	1
**Neurological examination**			
Romberg sign [*n* (%)]	0 (0%)	0 (0%)	1
DTR reduced/absent [*n* (%)]	15 (71%)	6 (19%)	**<0.001**
Sensory exam. [*n* (%)]	20 (95%)	28 (88%)	0.578
Normal			
Reduced vibration detection	1 (5%)	3 (9%)	
↓ Pinprick	0 (0%)	1 (3%)	
Clinical myotonia [*n* (%)]	17 (81%)	5 (16%)	**<0.001**
MIRS [*n* (%)]	1 (5%)	n.p.	n.a.
No impairment	5 (24%)		
Minimal signs	6 (29%)		
Distal weakness	8 (38%)		
Mild/moderate proximal weakness	1 (5%)		
Severe proximal weakness			
Current pain medication [*n* (%)]	3 (14%)	11 (34%)	0.125

DM1, myotonic dystrophy type 1; DM2, myotonic dystrophy type 2; IQR, interquartile range; DTR, deep tendon reflexes; Sensory exam., sensory examination; MIRS, muscle impairment rating scale; group comparison by Mann–Whitney U test for continuous, by Chi^2^-test for nominal variables with three or more categories and by Fisher-test for dichotomous variables; *p*-values < 0.05 printed in bold.

The most commonly reported multisystemic involvements were daytime sleepiness, hypertension and dyslipidemia. Daytime sleepiness affected more DM1 than DM2 patients (Fisher-test *p* = 0.035), and more DM2 than DM1 suffered from hypertension and dyslipidemia (*p* < 0.001 and *p* = 0.008, respectively; Table 1). Differences in proportions of patients with hypertension remained significant also after adjustment for age and gender.

On neurological examination, mild signs of sensory deficits were observed in a few patients only (1 DM1 and 4 DM2). The Romberg test was negative in all patients. More DM1 than DM2 patients showed reduced or absent deep tendon reflexes (DTRs) (*p* < 0.001) as well as myotonia (*p* < 0.001). These differences remained significant after adjustment for age and gender (Supplementary Table1) and for BMI and gender (Supplementary Table 2), respectively. In line with the inclusion criteria, none of the patients and none of the healthy controls showed signs of polyneuropathy according to NCS (Supplementary Table 3).

### 3.2 Characteristics of pain

All but one DM1 patient and all DM2 patients completed the pain questionnaires (Table 2). Most patients reported multiple pain sites: The thighs were the most frequent pain site in both groups. Overall, pain severity was similar in both groups. However, DM2 patients reported stronger pain during the examination (*p* = 0.037) and had higher BPI pain interference scores than DM1 (*p* = 0.012). Accordingly, slightly more DM2 than DM1 patients were currently taking pain medications, although the difference was not statistically significant (34% vs. 14%, *p* = 0.125, Table 1). NSAIDs (*n* = 5) and pain-modulating drugs (pregabalalin and gabapentin, *n* = 4) were most commonly used. Adjusted analyses suggest that these differences were related to the older age of DM2 patients (Supplementary Table 1). In both groups, pain was most often described as “tiring” and “cramping” (in over 75% of DM1 and DM2 patients) followed by “tearing,” “sharp,” “burning” and “unbearable.”

**Table 2:** Pain features.

Variable	DM1	DM2	*p*-value
	*n* = 20	*n* = 32	(DM1 vs DM2)
**Brief pain inventory (BPI)**			
**Pain site [*n* (%)]**			
Head	9 (45)	17 (53)	0.776
Back	12 (60)	17 (53)	0.776
Upper limb proximal	8 (40)	12 (38)	1.000
Upper limb distal	12 (60)	11 (34)	0.090
Lower limb proximal	14 (70)	27 (84)	0.299
Lower limb distal	13 (65)	18 (56)	0.575
Pain last week [*n* (%)]	20 (95%)	32 (100%)	0.396
Pain today [*n* (%)]	13 (62%)	26 (81%)	0.202
**Pain intensity last 24 h, NRS 0–10**			
Worst (median [IQR])	6.0 [3.3; 7.0]	6.0 [4.0; 8.0]	0.550
Average (median [IQR])	3.5 [3.0; 5.0]	4.0 [3.0; 5.0]	0.696
Mildest (median [IQR])	2.0 [0.3; 3.8]	2.0 [0.3; 4.0]	0.811
Present (median [IQR])	1.0 [0.0; 3.8]	4.0 [1.3; 5.0]	**0.037**
Pain severity (median [IQR])	3.3 [2.1; 4.7]	4.3 [2.5; 5.4]	0.318
Pain interference (median [IQR])	2.1 [1.5; 3.4]	4.1 [2.6; 5.6]	**0.012**
**Pain DETECT**			
**Pain pattern [*n* (%)]**			
Permanent pain	5 (25%)	12 (38%)	**0.035**
Permanent pain + attacks	1 (5%)	10 (31%)	
Pain attacks, otherwise free of pain	11 (55%)	8 (25%)	
Attacks, no complete remission	3 (15%)	2 (6%)	
**Radiating pain [*n* (%)]**			
Neuropathic pain quality sum score (median [IQR])	5 (25%)	25 (78%)	**<0.001**
Final score (median [IQR])	7.5 [5.0; 13.5]	10.5 [7.3; 17.8]	0.132
**Neuropathic pain [*n* (%)]**	9.0 [5.3–14.5]	12.5 [9.0–19.8]	0.094
Unlikely	14 (70%)	16 (50%)	0.112
Possibly	4 (20%)	7 (22%)	
Likely	2 (10%)	9 (28%)	
**Pain disability index (PDI)**			
Sumscore 0–70 (median [IQR])	17.0 [9.3–23.0]	26.0 [15.5–39.0]	**0.026**

DM1, myotonic dystrophy type 1; DM2, myotonic dystrophy type 2; IQR, interquartile range; NRS, numeric rating scale (0–10); group comparison by Mann–Whitney U test for continuous, by Chi^2^-test for nominal variables with three or more categories and by Fisher-test for dichotomous variables; *p*-values < 0.05 printed in bold.

The Pain-DETECT questionnaire allowed for a specific characterization of the temporal pain patterns and neuropathic pain characteristics. Its total score indicates the probability that patients suffer from neuropathic pain. A striking difference between DM1 and DM2 patients was observed with regard to the temporal pain pattern. More DM2 than DM1 patients reported permanent pain without attacks or permanent pain with worsening attacks, whereas more DM1 than DM2 patients experienced pain attacks with or without complete remission” (*p* = 0.035). Furthermore, radiating pain was reported by over three-quarters of DM2 patients but only by one-quarter of DM1 patients (*p* < 0.001). In contrast, the sum score for neuropathic pain characteristics did not differ between patient groups. Consequently, the Pain-DETECT final score was only marginally higher in DM2 than DM1 patients (*p* = 0.094) as was the estimated likelihood for a neuropathic pain component: unlikely 70% DM1 vs. 50% DM2, possible 20% DM1 vs. 22% DM2, likely 10% DM1 vs. 28% DM2 (*p* = 0.112). These results were not affected by adjustment for gender and age nor by adjustment for gender and BMI (Supplementary Tables 1, 2). However, the Pain DETECT sum score for neuropathic pain characteristics, the Pain DETECT final score and the likelihood for neuropathic pain according to the Pain DETECT questionnaire also increased with age (Supplementary Table 1).

In line with the BPI pain interference score, the PDI indicated that DM2 patients experienced higher degrees of pain-related disability in their everyday activities than DM1 patients (*p* = 0.026), which could again be linked to the older age in the DM2 group (Supplementary Table 1). In both patient groups, strong correlations were observed between the BPI pain severity and interference score (DM1 rs 0.683 *p* < 0.001, DM2 rs 0.721 *p* < 0.001) as well as between the BPI pain interference score and the PDI (DM1 rs 0.804 p < 0.001, DM2 rs 0.761 *p* < 0.001). In contrast, only within the DM2 cohort, the Pain-DETECT final score correlated with the BPI pain interference score as well as with the PDI (rs 0.35, *p* = 0.047 and rs 0.472, *p* = 0.006, respectively).

### 3.3 Quantitative sensory testing

**Figure 1:**
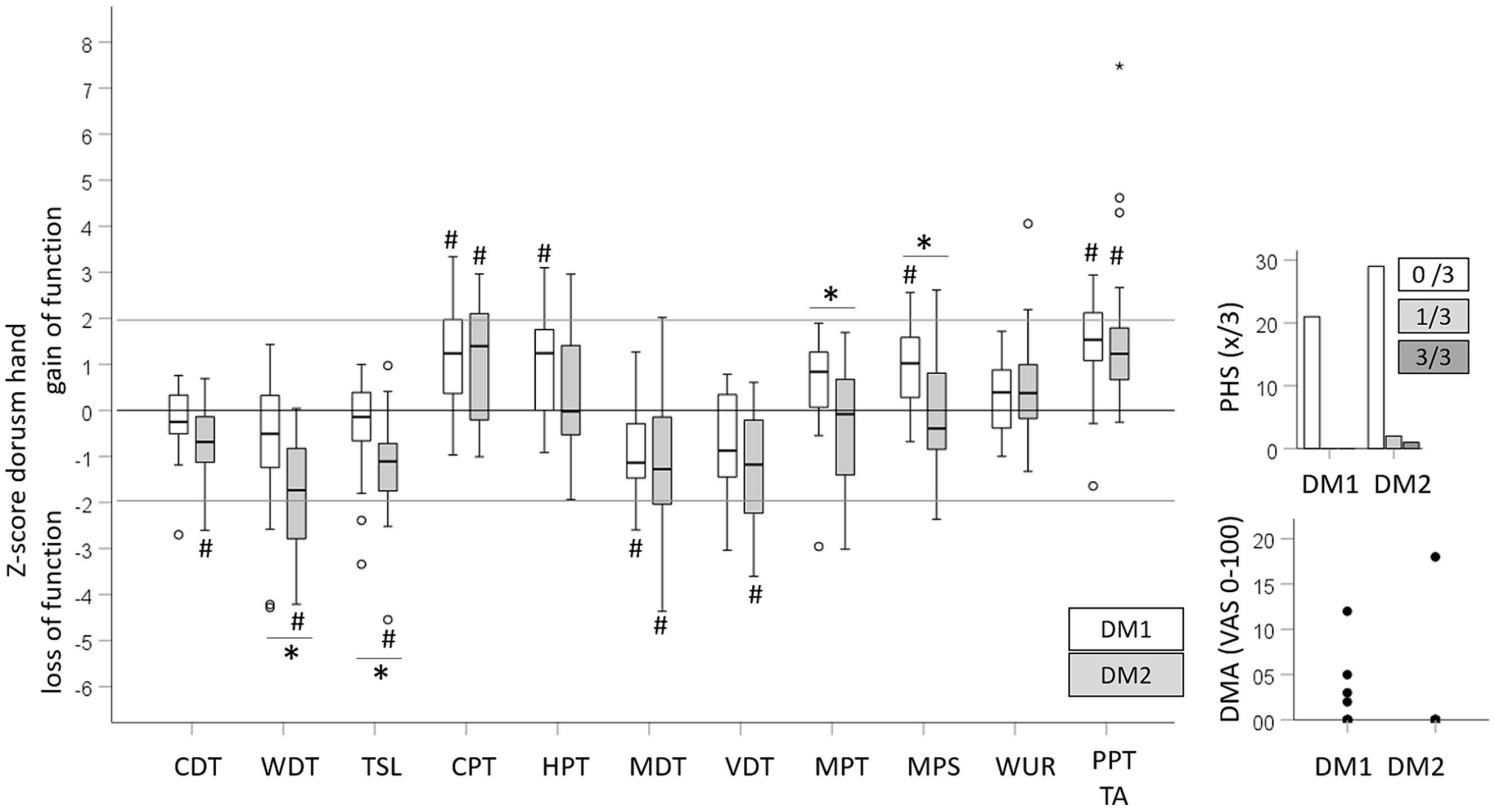
Sensory profiles at the dorsum of the hand. Z-scores were calculated for all QST parameters for DM1 and DM2 patients based on the DFNS age- and gender-specific reference data. Denoted group difference corresponds to the statistical comparisons depicted in Table 2. ^*^Significant difference between DM1 and DM2 patients; ^#^significantly different from the control group.

**Figure 2:**
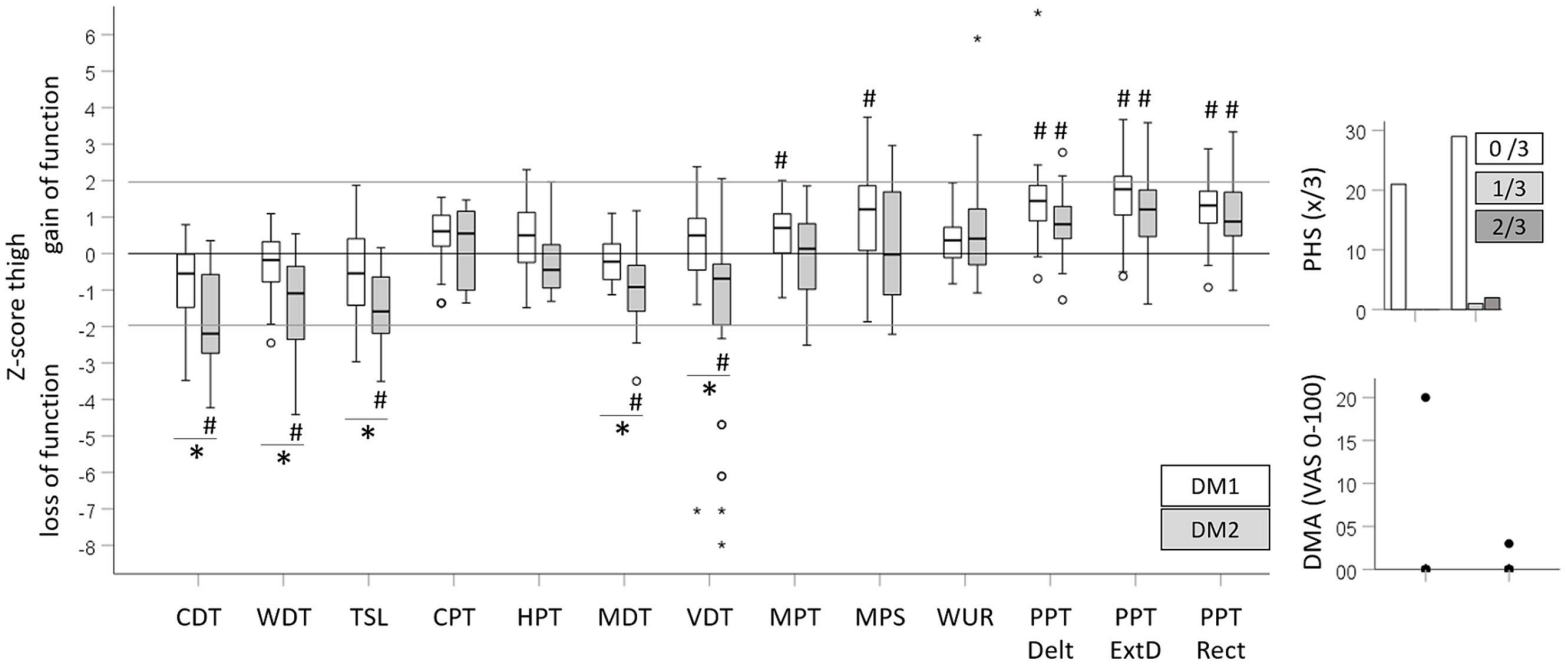
Sensory profiles at the thigh. Z-scores were calculated for all QST parameters for DM1 and DM2 patients on the basis of the age- and gender-matched healthy control group. De noted group difference corresponds to the statistical comparisons depicted in Table 2. ^*^Significant difference between DM1 and DM2 patients; ^#^significantly different from the control group.

Raw data of quantitative sensory testing (QST) performed in all recruited patients and controls are depicted in Table 2. Figure 1 shows the sensory profiles of DM1 and DM 2 patients at the dorsum of the hand, with z-scores calculated based on DFNS reference values and Figure 2 shows the sensory profiles of both patient groups at the thigh, with z-scores calculated based on the healthy control group.

Impaired mechanical detection characterized the sensory profile of DM2 patients. At both testing sites, the CDT, WDT, TSL, MDT and VDT indicated a significant loss of function in DM2 patients compared to healthy controls or DFNS reference data, respectively. DM1 patients only showed a loss of function for the MDT at the dorsum of the hand, and a trend toward a loss in cold detection (CDT) at the thigh became significant after covariate adjustment (Supplementary Tables 1, 2). The more pronounced loss of function in DM2 than DM1 patients was reflected in the unadjusted (Table 3) and adjusted analysis (Supplementary Tables 1, 2). Independently, increasing age and BMI were associated with a loss for WDT, CDT and TSL. Conversely, reduced thermal and mechanical pain thresholds (gain of function) characterized the sensory profile of DM1 patients. DM1 patients showed significant cold and heat hyperalgesia (gain of function for the CPT and HPT) at the dorsum of the hand, while DM2 patients only showed cold hyperalgesia at the dorsum of the hand. Significant but clinically negligible mechanical hyperalgesia was observed in DM1 patients at both testing sites, with a gain of function for the MPT and MPS at the thigh and for MPS at the dorsum of the hand. In DM2 patients, the MPT and the MPS were not different from the control group or the reference data. Both patient cohorts showed pronounced pressure hyperalgesia at all testing sites with a trend toward a larger gain of function in DM1 patients. The differences in thermal and mechanical pain thresholds between patient cohorts, healthy controls, or reference data, respectively, were confirmed in the adjusted analyses (Supplementary Tables 1, 2). Furthermore, age was associated with higher HPT. Neither of the patient cohorts showed an elevated WUR and PHS occurred in three DM2 cases only. Equally, few patients showed DMA with low pain levels induced by light stroking (Table 3).

**Table 3:** Quantitative sensory testing.

					Comparison to reference data after z-transformation							Comparison to matched controls			
		Reference	DM1	DM2	KW-test overall	DM1 vs. CG	DM2 vs. CG	DM1 vs. DM2	Control	DM1	DM2	KW-test overall	DM1 vs. CG	DM2 vs. CG	DM1 vs. DM2
		Dorsum of the hand							Thigh (PPT at indicated measure sites)						
CDT, Δ °C from 32°C (median [IQR])		−1.3 [−1.9; −0.9]	−1.5 [−1.7; −0.9]	−2.1 [−2.7; −1.4]	**0.015**	0.668	**0.012**	0.063	−1.6 [−2.2; −1.3]	−2.0 [−3.3; −1.6]	−4.0 [−5.0; −2.0]	**<0.001**	0.129	**<0.001**	**0.030**
WDT, Δ °C from 32°C (median [IQR])		2.0 [1.4; 2.8]	2.3 [1.4; 3.9]	4.7 [3.1; 7.9]	**<0.001**	0.163	**<0.001**	**0.004**	2.9 [2.3; 3.6]	3.0 [2.4; 3.8]	4.3 [3.2; 7.0]	**<0.001**	1.000	**<0.001**	**0.009**
TSL, Δ °C CDT/WDT (median [IQR])		3.1 [2.1; 4.5]	2.7 [2.1; 4.5]	6.0 [4.7; 9.1]	**<0.001**	0.539	**<0.001**	**0.002**	4.3 [3.1; 5.5]	5.4 [3.4; 8.0]	8.6 [5.6; 11.2]	**<0.001**	0.447	**<0.001**	**0.009**
PHS, x/3 [n (%)]	0/3	27 (100%)	21 (100%)	29 (91%)	0.099				27 (100%)	21 (100%)	29 (91%)	0.099			
	1/3	0 (0%)	0 (0%)	2 (6%)					0 (0%)	0 (0%)	1 (3%)				
	2/3	0 (0%)	0 (0%)	0 (0%)					0 (0%)	0 (0%)	2 (6%)				
	3/3	0 (0%)	0 (0%)	1 (3%)					0 (0%)	0 (0%)	0 (0%)				
CPT, °C (median [IQR])		10.3 [5.0; 15.6]	21.4 [16.2; 27.6]	21.2 [8.4; 26.0]	**0.001**	**0.003**	**0.002**	1.000	12.0 [3.8; 24.7]	20.2 [12.4; 25.0]	19.6 [3.1; 25.9]	0.526			
HPT, °C (median [IQR])		44.6 [42.6; 46.6]	40.5 [37.9; 43.3]	45.1 [41.3; 46.8]	**0.017**	**0.009**	0.539	0.053	44.6 [40.0; 46.9]	41.6 [39.1; 45.1]	45.2 [42.3; 47.2]	0.073			
MDT, mN (median [IQR])		1.3 [0.7; 2.5]	2.1 [0.8; 4.6]	4.2 [1.7; 9.3]	**0.001**	**0.020**	**<0.001**	0.712	3.0 [1.9; 7.0]	4.0 [2.4; 6.5]	8.0 [4.2; 17.0]	**0.002**	1.000	**0.003**	**0.038**
VDT, x/8 (median [IQR])		7.7 [7.4; 8.0]	7.4 [7.0; 8.0]	7.1 [6.8; 7.6]	**0.001**	0.082	**<0.001**	0.253	6.3 [5.9; 6.7]	6.7 [6.0; 7.0]	5.8 [4.9; 6.2]	**0.002**	1.000	**0.031**	**0.002**
MPT, mN (median [IQR])		78.0 [44.7; 136.3]	48.5 [23.8; 87.5]	93.8 [48.5; 254.3]	**0.010**	0.057	0.524	**0.005**	90.5 [42.2; 147.0]	39.4 [25.2; 79.0]	66.3 [33.9; 181.0]	**0.023**	**0.028**	1.000	0.074
MPS, NRS 0–100 (median [IQR])		1.4 [0.7; 2.7]	1.7 [0.5; 3.2]	0.4 [0.2; 1.3]	**0.006**	**0.017**	0.761	**0.004**	0.4 [0.2; 0.7]	1.3 [0.5; 2.6]	0.4 [0.1; 1.9]	**0.017**	**0.018**	1.000	0.080
DMA [*n* (%)]		0 (0%)	4 (19%)	1 (3%)	0.444				0 (0%)	1 (5%)	1 (3%)	0.553			
max, VAS 0–100			0.12	0.18						0.2	0.03				
WUR, NRS ratio (median [IQR])		2.1 [1.5; 3.1]	2.7 [1.8; 3.6]	2.5 [1.9; 3.6]	0.181				2.7 [2.0; 4.2]	3.4 [2.6; 4.3]	3.5 [2.4; 5.8]	0.200			
PPT TA, kPa (median [IQR])		455.5 [377.7; 549.4]	248.1 [224.6; 365.8]	343.2 [288.3; 401.8]	**<0.001**	**<0.001**	**<0.001**	0.601	448.2 [346.2; 598.2]	248.1 [224.6; 365.8]	343.2 [288.3; 401.8]	**<0.001**	**0.000**	**0.009**	0.156
PPT Delt, kPa (median [IQR])									390.3 [277.5; 470.7]	215.7 [166.7; 292.7]	285.9 [226.5; 341.5]	**<0.001**	**0.000**	**0.017**	0.159
PPT Rectfem, kPa (median [IQR])									414.8 [310.9; 693.3]	242.2 [201.0; 306.0]	295.7 [202.5; 352.3]	**<0.001**	**0.000**	**0.004**	0.703
PPT ExtDig, kPa (median [IQR])									350.1 [257.9; 493.3]	173.6 [147.1; 237.3]	218.7 [174.3; 299.3]	**<0.001**	**0.000**	**0.001**	0.306

DM1, myotonic dystrophy type 1; DM2, myotonic dystrophy type 2; CG, control group; IQR, interquartile range; CDT, cold detection threshold; WDT, warm detection threshold; TSL, thermal sensory limen; CPT, cold pain threshold; HPT, heat pain threshold; MDT, mechanical detection threshold; VDT, vibration detection threshold; MPT, mechanical pain threshold; MPS, mechanical pain sensitivity; WUR, wind-up ratio; PPT, pressure pain threshold; TA, thenar muscle; Delt, deltoid muscle; Rectfem, rectus femoris muscle; ExtDig, extensor digitorum communis muscle; group comparison by Kruskal-Wallis test with Dunn’s post-hoc test for continuous, by Chi^2^-test for nominal variables with three or more categories and by Fisher-test for dichotomous variables; *p*-values < 0.05 printed in bold.

Overall, QST parameters correlated poorly with results in the Pain DETECT questionnaire which quantifies neuropathic pain symptoms (Table 4). In DM2 patients, higher Pain DETECT final scores were significantly correlated with a loss in warm detection (WDT) at the dorsum of the hand and a gain in mechanical pain perception (MPT and MPS) at the thigh. These correlations were moderate and would not withstand correction for multiple testing.

**Table 4:** Correlation between QST parameters and Pain DETECT final score.

		Correlation between Pain DETECT final score and z-scores of QST parameters at the dorsum of the hand			Correlation between Pain DETECT final score and z-scores of QST parameters at the thigh		
		DM1 and DM2	DM1	DM2	DM1 and DM2	DM1	DM2
CDT	rS	−0.164	−0.202	0.028	−0.131	−0.026	−0.005
	*p*-value	0.245	0.392	0.879	0.356	0.913	0.978
WDT	rS	**−0.426**	−0.189	**−0.369**	**−0.287**	−0.338	−0.073
	*p*-value	**0.002**	0.426	**0.037**	**0.039**	0.145	0.692
TSL	rS	**−0.326**	−0.179	−0.209	−0.123	−0.152	0.094
	*p*-value	**0.018**	0.451	0.250	0.384	0.521	0.608
CPT	rS	0.191	0.444	0.018	0.149	0.208	0.160
	*p*-value	0.176	0.050	0.922	0.291	0.379	0.382
HPT	rS	−0.130	0.138	−0.170	−0.079	0.130	0.005
	*p*-value	0.359	0.562	0.353	0.577	0.586	0.980
MDT	rS	0.094	0.106	0.208	0.011	0.248	0.140
	*p*-value	0.507	0.658	0.254	0.938	0.293	0.445
VDT	rS	0.177	0.142	0.256	−0.083	0.002	0.002
	*p*-value	0.209	0.551	0.158	0.559	0.995	0.993
MPT	rS	−0.027	−0.092	0.207	0.122	−0.089	**0.421**
	*p*-value	0.849	0.700	0.255	0.387	0.709	**0.016**
MPS	rS	0.052	0.059	0.228	0.180	0.117	**0.377**
	*p*-value	0.714	0.805	0.210	0.201	0.623	**0.033**
WUR	rS	0.033	0.003	0.000	−0.024	−0.037	−0.109
	*p*-value	0.815	0.990	0.999	0.869	0.876	0.553
PPT TA	rS	−0.210	−0.408	0.077			
	*p*-value	0.135	0.074	0.676			
PPT Delt	rS				**−0.316**	−0.097	−0.268
	*p*-value				**0.023**	0.685	0.138
PPT Rectfem	rS				−0.207	0.064	−0.248
	*p*-value				0.141	0.788	0.172
PPT ExtDig	rS				−0.162	−0.005	−0.130
	*p*-value				0.252	0.984	0.479

DM1, myotonic dystrophy type 1; DM2, myotonic dystrophy type 2; CDT, cold detection threshold; WDT, warm detection threshold; TSL, thermal sensory limen; CPT, cold pain threshold; HPT, heat pain threshold; MDT, mechanical detection threshold; VDT, vibration detection threshold; MPT, mechanical pain threshold; MPS, mechanical pain sensitivity; WUR, wind-up ratio; PPT, pressure pain threshold; TA, thenar muscle; Delt, deltoid muscle; Rectfem, rectus femoris muscle; ExtDig, extensor digitorum communis muscle; rS, correlation coefficients according to Spearman; *p*-values < 0.05 printed in bold.

### 3.4 Intraepidermal nerve fiber density

Skin punch biopsies were performed in 52 of the 53 included DM patients. After completing all other assessments, one DM1 patient refused to provide a skin biopsy. Out of the 52 performed biopsies, the intraepidermal nerve fiber density (IENFD) could be reliably evaluated in 19 out of 21 DM1 patients and in 28 out of 32 DM2 patients. In 6 biopsies, processing and technical issues clearly affected the staining quality and compromised the evaluation. These were excluded from the analysis.

IENFD at both biopsy sites as well as the ratio between the proximal and the distal IENFD did not differ between DM1 and DM2 patient (Table 5). 63% of DM1 and 50% of DM2 patients showed a reduced distal IENFD compared to age- and sex-adjusted normal values. 64% of those patients with reduced distal IENFD also showed an IENFD proximal/distal ratio over 2.5 indicating a length-dependent pathology. An exclusive reduction of the proximal IENFD (in the presence of normal distal IENFD) was not observed. The proximal IENFD was lower among patients with reduced distal IENFD than among those with normal distal IENFD (median [IQR] 8.2 [4.8; 9.1] vs. 10.5 [7.4; 13.6] *p* = 0.018).

**Table 5:** Intraepidermal nerve fiber density.

Intraepidermal nerve fiber density (IENFD, fibers per mm)			
	DM1	DM2	*p*-value
	*n* = 21	*n* = 32	(DM1 vs. DM2)
Distal (median [IQR])	4.7 [3.8; 6.2]	4.0 [1.8; 7.9]	0.812
Proximal (median [IQR])	10.8 [7.1; 13.1]	8.1 [6.3; 10.4]	0.107
Ratio proximal/ distal (median [IQR])	1.9 [1.4; 3.0]	2.2 [1.3; 3.6]	0.884
Low distal IENFD [*n* (%)]	12 (63%)	14 (50%)	0.551

DM1, myotonic dystrophy type 1; DM2, myotonic dystrophy type 2; IQR, interquartile range; low distal IENFD, IENFD values below the 5%-quantile of established age and gender specific reference values (19); group comparison by Mann–Whitney U test for continuous and by Fisher-test for dichotomous variables.

Patients with a distal IENFD below the reference values did not differ from those with a distal IENFD in the normal range with regard to QST parameters at both measure sites, disease duration and questionnaire results (Table 6). Given the large number of comparisons, the number of significant results were in the expected range of false positives due to multiple testing.

**Table 6:** Correlation of reduced IENFD to QST parameters and questionnaire results.

	DM1 and DM2			DM1			DM2		
	IENFD low *n* = 21	IENFD normal *n* = 26	*p*-value	IENFD low *n* = 12	IENFD normal *n* = 7	*p*-value	IENFD low *n* = 14	IENFD normal *n* = 14	*p*-value
**Z-scores of QST parameters at the dorsum of the hand (median [IQR])**									
CDT	−0.3 [−0.5; 0.3]	−0.9 [−1.2; −0.1]	0.061	−0.2 [−0.4; 0.2]	−0.3 [−1.2; 0.5]	0.672	−0.5 [−1.1; 0.4]	−0.9 [−1.3; −0.2]	0.141
WDT	−1.0 [−2.0; −0.3]	−1.4 [−3.1; −0.2]	0.740	−0.4 [−1.0; 0.2]	−0.1 [−2.6; 0.8]	0.933	−1.7 [−2.9; −1.0]	−1.5 [−3.6; −0.8]	0.765
TSL	−0.5 [−1.4; −0.1]	−1.1 [−1.8; −0.2]	0.386	−0.2 [−0.4; 0.3]	−0.1 [−1.8; 0.6]	0.933	−0.9 [−1.8; −0.5]	−1.1 [−1.8; −0.8]	0.613
CPT	0.6 [0.0; 1.9]	1.6 [0.3; 2.1]	0.299	0.5 [0.1; 2.0]	1.7 [1.0; 2.7]	0.076	0.8 [−0.4; 2.0]	1.4 [−0.5; 2.0]	0.872
HPT	0.5 [−0.4; 1.9]	0.4 [−0.4; 1.5]	0.814	0.7 [−0.3; 1.9]	1.4 [1.2; 2.1]	0.205	0.2 [−0.9; 1.9]	−0.2 [−0.7; 1.1]	0.435
MPT	0.2 [−0.8; 0.9]	0.0 [−1.3; 0.9]	0.700	0.2 [0.0; 1.6]	0.9 [0.0; 1.6]	0.673	−0.1 [−1.6; 0.9]	−0.5 [−1.6; 0.6]	0.550
MPS	0.7 [−0.7; 1.2]	0.3 [−0.8; 1.3]	0.600	0.9 [−0.5; 1.5]	1.5 [0.7; 2.0]	0.151	0.4 [−1.0; 0.9]	−0.7 [−1.1; 0.6]	0.280
WUR	0.1 [−0.4; 0.9]	0.8 [0.3; 1.0]	0.054	−0.1 [−0.5; 0.9]	0.5 [0.4; 1.6]	0.205	0.2 [−0.3; 0.9]	0.8 [0.1; 1.0]	0.154
MDT	−1.2 [−1.6; −0.1]	−1.1 [−2.5; 0.3]	1.000	−1.1 [−1.5; −0.2]	−1.1 [−2.6; 0.6]	1.000	−1.3 [−2.2; −0.1]	−1.1 [−2.5; 0.3]	0.854
VDT	−1.1 [−1.9; 0.0]	−0.9 [−2.0; −0.2]	0.898	−0.5 [−1.5; 0.4]	−0.9 [−1.8; −0.2]	0.554	−1.3 [−2.2; −0.7]	−0.9 [−2.4; −0.1]	0.408
PPT TA	1.6 [1.0; 2.6]	1.1 [0.7; 2.0]	0.146	1.5 [0.9; 2.5]	2.0 [0.7; 2.2]	0.866	1.7 [1.2; 2.6]	1.0 [0.6; 1.3]	0.054
**Z-scores of QST parameters at the thigh (PPT at indicated measure sites, median [IQR])**									
CDT	−1.0 [−2.2; 0.1]	−1.9 [−2.9; −0.3]	0.121	−0.5 [−1.4; 0.2]	−0.2 [−2.0; 0.0]	0.397	−1.7 [−2.5; 0.0]	−2.3 [−3.0; −0.5]	0.334
WDT	−0.5 [−1.8; 0.1]	−1.2 [−2.0; −0.1]	0.422	−0.1 [−0.6; 0.8]	−0.5 [−1.8; 0.0]	0.091	−1.3 [−3.1; −0.3]	−1.3 [−2.1; −0.1]	0.408
TSL	−0.9 [−2.0; −0.3]	−1.6 [−2.1; −0.4]	0.392	−0.5 [−1.3; 0.6]	−0.9 [−1.7; 0.4]	0.447	−1.5 [−2.5; −0.6]	−1.7 [−2.1; −0.9]	0.927
CPT	0.3 [−1.0; 1.1]	0.7 [−0.2; 1.3]	0.173	0.3 [−0.7; 0.9]	1.1 [0.7; 1.3]	**0.022**	0.6 [−1.3; 1.2]	0.3 [−0.7; 1.2]	0.729
HPT	−0.2 [−1.0; 1.2]	0.1 [−0.6; 0.9]	0.507	0.5 [−0.9; 1.5]	0.4 [−0.2; 1.1]	0.866	−0.7 [−1.0; 0.1]	−0.2 [−0.6; 0.5]	0.291
MPT	0.4 [−0.3; 1.4]	0.5 [−0.9; 1.5]	0.881	0.9 [0.0; 1.3]	1.2 [0.4; 2.3]	0.175	0.2 [−1.0; 1.5]	0.2 [−1.5; 0.9]	0.662
MPS	0.8 [−0.4; 1.8]	0.4 [−1.1; 1.7]	0.645	0.7 [0.1; 1.8]	1.6 [0.4; 3.3]	0.272	0.8 [−1.0; 1.9]	−0.4 [−1.4; 1.4]	0.346
WUR	0.5 [−0.5; 0.8]	0.1 [−0.4; 1.7]	0.549	0.5 [−0.6; 0.7]	0.1 [−0.3; 0.6]	0.800	0.4 [−0.5; 0.8]	0.6 [−0.4; 2.0]	0.462
MDT	−0.7 [−1.0; 0.3]	−0.6 [−1.2; 0.0]	0.684	−0.2 [−0.7; 0.5]	−0.2 [−0.9; −0.1]	0.374	−0.8 [−1.5; −0.5]	−0.9 [−1.8; 0.3]	0.872
VDT	−0.6 [−1.7; 0.5]	−0.6 [−1.7; 0.4]	0.772	0.4 [−0.4; 0.9]	0.0 [−0.6; 1.0]	0.899	−0.8 [−3.0; −0.5]	−1.3 [−2.6; −0.4]	0.963
PPT Delt	0.9 [0.5; 1.6]	0.8 [0.3; 1.6]	0.806	1.3 [0.8; 1.7]	1.2 [0.3; 1.8]	0.672	0.8 [0.3; 1.2]	0.8 [0.3; 1.5]	0.679
PPT Rectfem	1.1 [0.4; 1.6]	1.0 [0.6; 1.8]	0.814	1.3 [0.7; 1.6]	1.2 [0.5; 1.9]	0.933	0.8 [0.1; 1.7]	0.9 [0.6; 1.8]	0.646
PPT ExtDig	1.4 [0.5; 1.9]	1.4 [0.6; 2.0]	0.923	1.5 [0.4; 2.0]	1.6 [1.0; 2.2]	0.374	1.4 [0.5; 1.7]	1.0 [0.3; 1.9]	0.520
**Time since DM onset and questionnaire data (median [IQR])**									
Time since onset, years	12.5 [7.0; 25.0]	15.0 [5.0; 21.0]	0.940	12.5 [7.8; 24.5]	11.0 [4.0; 19.0]	0.553	12.5 [6.8; 25.0]	18.0 [5.0; 27.5]	0.549
BPIpain interference	3.6 [2.1; 5.1]	3.4 [1.6; 6.2]	0.843	2.1 [1.4; 4.4]	2.1 [1.6; 3.1]	0.964	4.4 [2.9; 5.2]	4.4 [3.0; 6.6]	0.836
BPI pain severity	3.5 [2.5; 4.9]	4.5 [2.9; 5.3]	0.414	3.5 [2.3; 4.8]	3.0 [1.0; 4.5]	0.364	3.6 [2.5; 5.2]	4.9 [3.9; 5.9]	0.181
PDI sumscore	23.0 [13.5;33.5]	25.0 [14.5;40.0]	0.529	19.0 [9.0; 32.0]	15.0 [14.0; 20.0]	0.650	24.0 [14.8; 37.3]	31.5 [16.8; 43.3]	0.408
Pain DETECT final score	10.0 [5.5; 19.5]	12.0 [8.5; 15.0]	0.536	8.0 [2.0; 11.0]	15.0 [7.0; 17.0]	0.069	16.5 [8.3; 21.5]	10.5 [8.8; 14.3]	0.333

IENFD low, distal IENFD values below the 5%-quantile of established age and gender-specific reference values (19); DM1, myotonic dystrophy type 1; DM2, myotonic dystrophy type 2; CDT, cold detection threshold; WDT, warm detection threshold; TSL, thermal sensory limen; CPT, cold pain threshold; HPT, heat pain threshold; MDT, mechanical detection threshold; VDT, vibration detection threshold; MPT, mechanical pain threshold; MPS, mechanical pain sensitivity; WUR, wind-up ratio; PPT, pressure pain threshold; TA, thenar muscle; Delt, deltoid muscle; Rectfem, rectus femoris muscle; ExtDig, extensor digitorum communis muscle; NRS, numeric rating scale; IQR, interquartile range; BPI, brief pain inventory; PDI, pain disability index; statistical comparison by Mann–Whitney U test, *p*-values < 0.0.5 printed in bold.

In analogy to the Besta criteria for the diagnosis of small fiber neuropathy (21), DM patients with isolated signs of small fiber affection were identified. Eight patients (7 DM2 patients) presented significantly altered thermal thresholds on QST, pathologically reduced IENFD and fell into category 2 or 3 of the pain-DETECT predicting a high probability of having neuropathic pain. Six additional patients (3 DM2 patients) presented both reduced IENFD and altered thermal thresholds on QST, even though their clinical probability of having neuropathic pain according to the pain DETECT score was “unlikely” (category 1).

## 4 Discussion

According to the most recent IASP (International Association for the Study of Pain) classification, chronic pain can be classified into neuropathic, nociceptive, nociplastic and “mixed.” Correct classification of pain into one of these categories has relevant implications for its treatment. Yet, there is no agreement on the type of pain occurring in DM and its pathophysiology (3, 8, 10). This represents a critical unmet need, as chronic pain substantially compromises the mental and physical health of DM patients (5, 9). The results of our study confirm a neuropathic pain component contributing to both, DM1 and DM2 and particularly indicate small fiber affection. First, according to the pain characteristics evaluated in the Pain DETECT questionnaire (15), 30% of DM1 and 50% of DM2 are likely or at least possibly affected by neuropathic pain. Second, large part of both patient cohorts showed reduced IENFD at the distal leg (63% DM1 and 50% DM2) which is indicative for small nerve fiber deterioration (22). Third, sensory profiles evaluated by QST revealed two distinct yet equally prominent patterns that are characteristic for neuropathic pain conditions (23). Importantly QST results not only suggest small but also large fiber affection. One particular strength of our study is the direct comparison of DM1 and DM2 patients in comparison to healthy controls. Important differences and similarities between the DM1 and DM2 cohorts were observed. The possible implications of these findings are laid out in the following paragraphs.

### 4.1 Similar age adjusted pain interference and disability in DM1 and DM2 patients

Previously published studies on pain characteristics in DM addressed either DM1 or DM2 patients separately. Therefore, direct comparisons of pain prevalence, pain features and impact of pain were limited (5, 6, 10, 24). Our data confirm that pain features and severity do not significantly differ between DM1 and DM2 patients, who reported pain in similar body regions and used similar pain descriptors. The higher pain interference (BPI), the higher pain related disability (PDI) and the more common use of pain medication in DM2 could have been misinterpreted as a higher impact of pain and higher disease burden in DM2 patients, as also highlighted in the PRISM-2 study (24). However, our adjusted analyses show that older age of DM2 patients fully explained these differences in pain features. Accordingly, disease burden in DM1 and DM2 patients seem to be similar when age is taken into account. Higher pain sensitivity with increasing age has been demonstrated in experimental and clinical studies. It is thought to be related to physiological and anatomical changes occurring with aging (central sensitization, immune, neuroendocrine, inflammatory and autonomic changes) (25). Thus, confounding by age must be considered in the design of future clinical trials addressing pain in the DM population.

### 4.2 Different neuropathic pain mechanisms in DM1 and DM2 patients indicated by clinical pain characteristics and quantitative sensory testing

According to the Pain DETECT questionnaire in 30% of DM1 patients and in 50% of DM2 patients a neuropathic pain component appeared likely. Interestingly, DM1 patients reported recurrent pain attacks and exacerbations more often than DM2 patients, whose temporal pattern was more chronic and persistent. In contrast, radiating pain was reported more often by DM2 than DM1 patients. Thereby, the final Pain-DETECT score did not significantly differ between the two cohorts. The prominent but clinically distinct symptoms suggest a neuropathic pain component potentially related to different mechanisms in the two DM cohorts.

This is in line with the QST results that showed more gain in pain sensitivity in DM1 patients and a pronounced sensory loss in DM2 patients. The QST allowed us to explore the somatosensory function of our cohort better, assessing the large sensory nerve fibers and the thinly or unmyelinated C- and Aδ-fibers. It detects loss and/or gain of sensory function and finds application in assessing patients with neuropathic, nociceptive and nociplastic pain. Overall, we found a pronounced thermal (CDT, WDT, TSL) and mechanical detection loss (MDT, VDT) mainly in the DM2 population. This indicates an affection of small and large fiber function. In contrast, DM1 patients only showed a discreet loss in cold and mechanical detection, but were characterized by eminently reduced thermal and mechanical pain thresholds (gain of function). This suggests a more pronounced role of peripheral and central sensitization processes in DM1 patients which could be related to the more frequent pain attacks in DM1 patients due spontaneous firing of neurons of the pain pathway. In both patient groups, there was pressure hyperalgesia at all tested sites—more evident in DM1 patients. Reduced PPT previously observed in DM2 patients was interpreted as a result of sensitization of peripheral deep tissue nerve fibers in the presence of muscle dysfunction (8, 10). The more pronounced pressure hyperalgesia in DM1 patients might be related to the typically strong clinical myotonia in these patients. Additionally, both DM cohorts showed cold hyperalgesia at the dorsum of the hand which is likely related to the reduced function of cold sensitive nerve fibers indicated by the loss in cold detection (26, 27). The QST pattern observed in our cohort of DM2 patients has been observed in polyneuropathies and central pain conditions (23, 28), but also in chronic non-neuropathic pain syndromes (29). Our study found no apparent signs of central sensitization (normal WUR and absent allodynia). DM1 patients showed instead a tendency toward mixed thermal and mechanical loss with mechanical hyperalgesia, as described in peripheral neuropathies and other chronic pain conditions such as lower back pain (23, 28, 30). Neither DM1 nor DM2 patients showed an elevated WUR, and DMA was rare and mild. These are genuine signs of central sensitization that can but not necessarily occur in neuropathic pain states (23).

Comparison to previous QST studies in DM populations support our findings of an abnormal somatosensory function, but suggest that such a clear distinction between sensory profiles of DM1 and DM2 patient might not be an ironclad rule. In a recent study on 16 DM1 patients without diabetes mellitus a loss in warm and cold detection (WDT, CDT) was observed compared to healthy controls. In this study, only the thermal thresholds and no skin biopsies were performed, so correlations with reduced IENFD or pain questionnaires could not be analyzed (31). However, this observation (in contrast to our findings) suggests that pronounced signs of small fiber dysfunction can indeed also be present in DM1 patients and are not only characteristic for the DM2 population. The QST sensory profile of DM2 patients with myalgia depicted by Moshourab et al. revealed mainly a reduced pressure pain threshold, thus hypothesizing that myalgia could be caused by peripheral mechanisms within the muscle, as suggested by differences in the transcriptome profile of muscle biopsies between myalgia and non-myalgia patients (8). In line with our results, they also found a tendency toward loss of function for mechanical and thermal thresholds of unclear origin, suggesting a separate pain mechanism. They also observed a higher WUR and a higher MPS in DM2 patients with myalgia than in non-myalgic patients. This suggests that DM2 patients may indeed adopt these signs of central sensitization despite of the absence of mechanical hyperalgesia or elevated WUR in our patient cohort. However, the role of comorbidities such as diabetes, that was present in some patients in the study by Moshourab et al., warrants future research. In another study by Van Vliet et al. (10), reduced PPT was found in DM2 patients in comparison to healthy controls and higher PPT values compared to fibromyalgia patients. This study also evaluated electrical pain thresholds and conditioned pain modulation (CPM) which were not different between DM2 patients and healthy controls. In both previous studies fibromyalgia patients have also been used as controls (8, 10). The more prominent mechanical hyperalgesia among fibromyalgia patients than among DM2 patients underscores the less prominent role of central sensitization in the DM2 population. More detailed QST assessments, including conditioned pain modulation tests and comparisons to other patient populations (e.g., DM1 vs. fibromyalgia patients) should be considered in future studies.

Our QST results in particular the loss in sensory function could represent another manifestation of the progeroid process hypothesized to occur in the DM population (32). Particularly, impaired thermal, mechanical, and vibration detection are often observed in the elderly population (33). This is in line with recent results showing that defects in alternative splicing, a phenomenon associated with aging, occurs in patients with DM (34). Our results indicate that progeroid processes might affect predominantly small fibers responsible for thermal detection since age was associated with the loss in thermal but not in mechanical detection. In addition, a higher BMI further contributed to the loss in thermal detection in our patient cohorts. It is well known that obesity drives neuropathic processes (independent of diabetes) most likely through lipid signaling and salient inflammation (35, 36). At an early stage, a loss in sensory function may indicate the presence of a subclinical neuropathy, as demonstrated in a recent QST study on patients with pre-symptomatic familiar amyloidosis (37).

### 4.3 Reduced intraepidermal nerve fiber density in DM1 and DM2 patients

In both, DM1 and DM2 patients, the skin biopsy results showed high percentages (63% DM1 50% DM2) of reduced distal IENFD indicating small nerve fiber involvement. Importantly, the overall IENFD did not significantly differ between DM1 and DM2 patients, neither in the distal nor in the proximal skin biopsies. To our knowledge this is the first study that investigated the IENFD in DM2 patients. In a very recent study, Solbakken et al. (38) examined the IENFD, along with NCS and QST, in 20 DM1 patients. Their results match ours in that 50% of the patients showed pathologies of the peripheral nerves (small and/or large fibers). However, only 10% showed an IENFD below the normal range. Besides this study, we identified one conference abstract reporting on reduced IENFD in 3 of the 3 investigated DM1 patients and abnormal laser-evoked potentials in 2 of these patients (39).

How a reduced IENFD relates to the primary disease mechanisms needs to be further explored. Of particular interest could be a relationship with the number of CTG-repeats. A higher number of CTG repeats in the DMPK gene are known to be correlated with an earlier onset and a more severe phenotype of the disease in DM1 patients (2). DM transgenic mouse models (DMSXL) showed that transgenic mice carrying short repeat expansions (300–500 CTG) do not show any signs of peripheral nerve involvement. In contrast, mice with >1,300 CTG repeats show relevant axonopathy and neuronopathy (40). In their study on DM1, Solbakken et al. observed a trend toward a correlation of large CTG-repeats and large fiber damage as indicated by NCS (38) which had been reported before (41). However, Solbakken et al. found no evidence for a relationship between CTG-repeats and IENFD indicating small fiber damage. The small sample size and the low prevalence of pathologically reduced IENFD impede a firm conclusion. The role of repeat-associated non-ATG (RAN) translation and its potential interdependence with factors such as alternative splicing and proinflammatory mediators for the neuropathic pain component of myalgia in DM needs further exploration.

### 4.4 Little associations of clinical pain characteristics, IENFD and QST

Overall associations between clinical pain characteristics, IENFD and QST results were basically absent. Patients with a distal IENFD below the reference values did not differ from those with a distal IENFD in the normal range with regard to QST parameters, disease duration and questionnaire results. Therefore, the role of reduced IENFD as a cause of chronic pain remains unclear. Furthermore, the few correlations between the Pain DETECT final score and QST results were small and should be interpreted with caution. Based on the correlations of the Pain DETECT final score with more pronounced loss in warm detection and gain in mechanical pain thresholds (MPT, MPS) primarily in DM2 patients, one might argue carefully that small fiber dysfunction along with central sensitization might contribute to the neuropathic pain phenotype.

Inconsistencies in the correlation of the results between clinical pain characteristics, IENFD and QST are not uncommon in the literature. Based on this observation, the clinical diagnosis of small fiber neuropathy is recommended to be based on at least two indicators: clinical symptoms, abnormal thermal thresholds evaluated by QST and reduced IENFD (21, 42). In their study on DM1, Solbakken et al. (5) have observed little overlap between both, IENFD as well as NCS and QST results. As highlighted in a recent review, the relationship between the structure and function of small nerve fibers is often troublesome. Indeed, the quantification of IENFD depicts structural changes without predicting the entity of functional changes, as the remaining fibers can be normal, hypofunctional, or sensitized (43). Another example of this challenging clinical interpretation is the observation that denervation of the epidermis has been described both in chronic neuropathic pain conditions and patients with hereditary insensitivity to pain (44, 45). Furthermore, many neurological diseases, either without pain or without apparent peripheral nerve involvement, have been found to show reduced IENFD, such as amyotrophic lateral sclerosis, Parkinson’s disease, dementia with Lewy bodies and Pompe disease (18, 46). The same considerations account for QST. Our own research has shown only limited associations between QST results and neuropathic pain characteristics reported in questionnaires among patients with peripheral artery disease (47) and acute herpes zoster (48). Even more, loss in mechanical detection at non-affected body regions during acute herpes zoster are associated with the risk for post herpetic neuralgia (48, 49). This suggests that reduced IENFD and alterations in sensory profiles established by QST could indicate initial peripheral nerve fiber affection (43). During disease progression altered nerve fiber function and/or nerve fiber density might translate differently to clinical symptoms.

### 4.5 Possible subgroups of DM patients and clinical implications

At the individual level, some patients mainly in the DM2 cohort showed pure signs of small fiber neuropathy, given the combination of pathological QST, IENFD and clinical pain characteristics evaluated by the Pain DETECT questionnaire. At the same time, the loss in mechanical and vibration detection as evaluated by QST also suggests affection of large fiber function in both DM cohorts which is in line with the study by Moshourab et al. Dysfunction of sensory large fibers has been repeatedly reported in parallel to dysfunction of motor nerves. Back in 1978, a slowed motor nerve conduction velocity was observed, unrelated to glucose intolerance (50). A nerve biopsy study on randomly selected 13 DM patients identified a mild to moderate loss of myelinated fibers, suggesting the presence of axonopathy in at least some DM patients (51). Since then, studies suggesting peripheral nerve involvement in DM have increased (41, 52–54). Again, NCS showed little association with clinical symptoms (33).

Consequently, our findings indicate, in line with those by Solbakken et al. for DM1, the presence of subgroups within the well-known heterogeneity of the DM clinical spectrum. The examination of larger DM cohorts would be needed to identify different subgroups of patients with small fiber neuropathy, polyneuropathy, no neuropathic pain component and those with additional or isolated signs of central sensitization. Future research should also address the role of pain medications in the light of such subgroups and other factors such as CTG-repeats, physical activity, BMI, disability, cognitive involvement and depression.

Overall, the evident role of a neuropathic pain component supports the use of pain-modulating drugs (gabapentin, pregabalin, duloxetine, amitriptyline) and non-medical treatments targeting nerve fiber function in managing pain in DM patients. Furthermore, given that myotonia characterizes the clinical picture of DM to a varying extent, drugs like lamotrigine targeting both, neuropathic pain and myotonia, should be considered in patients who exhibit both, signs for a neuropathic pain component and strong myotonia.

### 4.6 Limitations

In addition to the relatively small DM patient cohorts, the limited investigation for alternative causes of peripheral nerve damage needs to be taken into account when interpreting our findings. Even though we have asked patients for the presence of multiple comorbidities that could cause an SFN (alcohol consumption, thyroid dysfunction, dyslipidaemia, rheumatoid arthritis, tumors in the past), not all causes of SFN have been systematically investigated (e.g., impaired glucose tolerance, vit. B12 deficiency, hyperuricaemia). Hypothyroidism as a possible cause for SFN has been only assessed anamnestically but not in blood. Most recruited patients are regularly followed up at our center and undergo laboratory control at least once a year, including thyroid function. Some recruited patients (5/20 DM1 and 12/32 DM2) had a known thyroid dysfunction under specific treatment. Another factor limiting the interpretation of correlations between IENFD and QST in our study is the choice of the hand dorsum instead of the foot dorsum for QST. Furthermore, the autonomic nervous system has not been investigated, so we cannot know whether the reduced IENFD might be causing autonomic symptoms or dysfunctions in these patients. As a final limitation, unfortunately, CTG repeat size related correlations could not be performed due to missing and very old reports on the CTG repeat size in our DM1 cohort.

## 5 Conclusion

Our results confirm that a neuropathic pain component contributes to the etiology of pain in patients with DM1 and DM2. Importantly, our finding extends the existing knowledge on DM by the identification of small fiber involvement. The IENFD was reduced in a substantial proportion of DM1 and DM2 patients. Sensory nerve fiber function seems to be differently affected in DM1 and DM2, with DM2 patients being characterized by impaired thermal and mechanical detection and DM1 patients most prominently by mechanical hyperalgesia. We observed little associations between QST results, IENFD and clinical pain characteristics. Therefore, altered nerve fiber function and reduced IENFD seem to be independent processes that might translate differently to clinical symptoms. In addition, our study proved that the pain of DM1 and DM2 patients differs less in quality and quantity than previously assumed. Both groups reported the same pain regions and qualities. Age as a likely confounder in previous studies should be accounted for in future research. Whether there is a connection to chronic pain and what role peripheral nerve damage plays in the pathophysiology of the diseases remains subject of future research. Here, RAN translation, alternative ion channel splicing and inflammatory factors may contribute to the origin of DM pain modulation. Finally, this growing evidence of neuropathic pain patterns in DM should be adopted in clinical diagnosis in order to appropriately adapt pain management strategies.

## Data availability statement

The original contributions presented in the study are included in the article/Supplementary material, further inquiries can be directed to the corresponding author.

## Ethics statement

The studies involving humans were approved by the Ethic Committee of the Ludwig-Maximilians-University Munich. The studies were conducted in accordance with the local legislation and institutional requirements. The participants provided their written informed consent to participate in this study.

## Author contributions

VS: Writing – original draft, Writing – review & editing, Data curation, Investigation. PB: Conceptualization, Data curation, Formal analysis, Investigation, Methodology, Writing – original draft, Writing – review & editing. AS: Conceptualization, Data curation, Methodology, Writing – review & editing. DI: Conceptualization, Data curation, Methodology, Writing – review & editing. BS: Conceptualization, Data curation, Methodology, Supervision, Writing – review & editing. FM: Conceptualization, Data curation, Funding acquisition, Investigation, Methodology, Writing – original draft, Writing – review & editing.

## Footnotes

^1^ https://www.dm-registry.org/de/index.de.html
