# Supplementary material for: Characterization of the neuropathic pain component contributing to myalgia in patients with myotonic dystrophy type 1 and 2
Source: Front Neurol. 2024 Aug 13;15:1414140. doi: 10.3389/fneur.2024.1414140 (PMC11347447; doi:10.3389/fneur.2024.1414140)
Supplement: Supplementary file 1 [file Table_1.docx]

**Supplementary table 1: Age and gender adjusted comparisons between DM2 and DM1 patients**

| **Linear GLM** | | | | |
| --- | --- | --- | --- | --- |
| **Outcome** | **Omnibus test p-value** | **Female vs  male (ref)** β [95%-CI] | **Age** β [95%-CI] | **DM2 vs DM1 (ref)** β [95%-CI] |
| Intraepidermal nerve fiber density (IENFD) per mm^2^ | | | | |
| proximal | 0.184 | -0.4 [-2.5; 1.7]  p=0.732 | -0.091 [-0.205; 0.023]  p=0.117 | -0.4 [-3.0; 2.2]  p=0.744 |
| distal | **0.043** | 0.9 [-0.5; 2.3]  p=0.201 | **-0.105 [-0.180; -0.031]**  **p=0.005** | 1.0 [-0.6; 2.6]  p=0.218 |
| ratio proximal/distal | 0.349 | -0.5 [-1.5; 0.6]  p=0.373 | 0.044 [-0.011; 0.100]  p=0.118 | -0.2 [-1.5; 1.0]  p=0.735 |
| Brief pain inventory (BPI) | | | | |
| Pain intensity last 24h, NRS | | | | |
| worst | 0.966 | 0.0 [-1.2; 1.3]  p=0.974 | 0.002 [-0.064; 0.068]  p=0.959 | 0.3 [-1.2; 1.9]  p=0.696 |
| average | 0.345 | 0.6 [-0.4; 1.6]  p=0.259 | 0.034 [-0.019; 0.087]  p=0.213 | -0.2 [-1.4; 1.1]  p=0.779 |
| mildest | 0.533 | 0.2 [-0.8; 1.1]  p=0.718 | 0.035 [-0.014; 0.085]  p=0.165 | -0.4 [-1.5; 0.8]  p=0.526 |
| present | 0.057 | 0.6 [-0.8; 1.9]  p=0.400 | 0.054 [-0.017; 0.124]  p=0.137 | 0.8 [-0.8; 2.5]  p=0.330 |
| Pain severity | 0.362 | 0.3 [-0.6; 1.3]  p=0.495 | 0.031 [-0.020; 0.082]  p=0.234 | 0.1 [-1.1; 1.3]  p=0.813 |
| Pain interference | **0.005** | 0.5 [-0.5; 1.5]  p=0.338 | **0.063 [0.009; 0.117]**  **p=0.022** | 0.6 [-0.6; 1.9]  p=0.313 |
| Pain DETECT | | | | |
| Neuropathic pain quality sum score | **0.038** | 0.5 [-3.1; 4.1]  p=0.785 | **0.247 [0.058; 0.436]**  **p=0.011** | -0.5 [-5.0; 3.9]  p=0.820 |
| Final score | **0.032** | 0.5 [-3.3; 4.3]  p=0.787 | **0.253 [0.057; 0.450]**  **p=0.011** | -0.2 [-4.8; 4.4]  p=0.935 |
| Pain disability index (PDI) | **0.018** | -1.1 [-9.0; 6.9]  p=0.795 | **0.476 [0.063; 0.889]**  **p=0.024** | 3.3 [-6.4; 13.0]  p=0.500 |
| **Ordinal logistic GLM** | | | | |
| **Outcome** | **Omnibus test p-value** | **F vs M(ref)** OR [95%-CI]  p-value | **Age** OR [95%-CI] p-value | **DM2 vs DM1 (ref)** OR [95%-CI] p-value |
| Pain DETECT | | | | |
| Pain pattern | 0.054 | 0.4 [0.1; 1.2]  p=0.099 | 1.0 [0.9; 1.1]  p=0.996 | 0.3 [0.1; 1.0]  p=0.053 |
| Neuropathic pain category | **0.014** | 0.6 [0.2; 2.0]  p=0.438 | **1.1 [1.0; 1.2]**  **p=0.011** | 0.9 [0.2; 3.5]  p=0.876 |
| **Binomial logistic GLM** |  |  |  |  |
| **Outcome** | **Omnibus test p-value** | **F vs M(ref)** OR [95%-CI]  p-value | **Age** OR [95%-CI] p-value | **DM2 vs DM1 (ref)** OR [95%-CI] p-value |
| Current pain medication | 0.397 | 1.3 [0.3; 4.8]  p=0.708 | 1.0 [0.9; 1.1]  p=0.916 | 3.0 [0.5; 16.6]  p=0.204 |
| Neurological examination | | | | |
| DTR reduced/absent | **0.001** | 0.4 [0.1; 1.7]  p=0.220 | 1.0 [0.9; 1.1]  p=0.905 | **0.1 [0.0; 0.4]**  **p=0.002** |
| Clinical myotoina | **<0.001** | 0.5 [0.1; 2.6]  p=0.406 | **0.9 [0.8; 1.0]**  **p=0.013** | **0.1 [0.0; 0.4]**  **p=0.003** |
| Brief pain inventory (BPI) |  |  |  |  |
| Pain today | **0.048** | **4.6 [1.2; 18.0]**  **p=0.028** | 1.0 [1.0; 1.1]  p=0.625 | 2.6 [0.6; 12.0]  p=0.211 |
| Pain DETECT – radiating pain | **<0.001** | 2.2 [0.6; 9.0]  p=0.260 | 1.0 [1.0; 1.1]  p=0.234 | **7.6 [1.6; 36.2]**  **p=0.011** |

DM1: myotonic dystrophy type 1; DM2: myotonic dystrophy type 2; NRS: numeric rating scale; DTR: deep tendon reflex; GLM: generalized linear model; β: regression coefficient; OR: odds ratio; 95%-CI: 95% confidence interval

| **Outcome** | **Omnibus test GLM p-value** | **F vs M(ref)** β [95%-CI]  p-value | **Age** β [95%-CI] p-value | **Overall group effect p-value** | **DM1 vs CG (ref)** β [95%-CI] p-value | **DM2 vs CG (ref)**  β [95%-CI] p-value | **DM2 vs DM1 (ref)** β [95%-CI] p-value |
| --- | --- | --- | --- | --- | --- | --- | --- |
| QST at the thigh and PPT at indicated measure sites | | | | | | | |
| CDT log | **<0.001** | -0.1 [-0.2; 0.0]  p=0.166 | **0.004 [0.000; 0.008]**  **p=0.038** | **<0.001** | **0.2 [0.1; 0.3]**  **p=0.004** | **0.3 [0.2; 0.4]**  **p<0.001** | **0.1 [0.0; 0.3]**  **p=0.033** |
| WDT log | **<0.001** | -0.1 [-0.2; 0.0]  p=0.039 | **0.005 [0.001; 0.008]**  **p=0.008** | **<0.001** | 0.1 [0.0; 0.2]  p=0.281 | **0.2 [0.1; 0.3]**  **p<0.001** | **0.1 [0.0; 0.2]**  **p=0.017** |
| TSL log | **<0.001** | -0.1 [-0.2; 0.0]  p=0.045 | **0.005 [0.001; 0.009]**  **p=0.006** | **<0.001** | **0.1 [0.0; 0.2]**  **p=0.020** | **0.3 [0.2; 0.4]**  **p<0.001** | **0.1 [0.0; 0.2]**  **p=0.038** |
| CPT | 0.378 | -3.3 [-7.9; 1.3]  p=0.158 | 0.056 [-0.145; 0.257]  p=0.585 | 0.353 | 4.3 [-1.6; 10.2]  p=0.149 | 1.7 [-3.7; 7.0]  p=0.538 | -2.7 [-8.8; 3.4]  p=0.393 |
| HPT | **0.009** | -0.6 [-2.2; 0.9]  p=0.422 | **0.093 [0.025; 0.162]**  **p=0.008** | 0.390 | -1.3 [-3.3; 0.8]  p=0.223 | 0.1 [-1.8; 1.9]  p=0.943 | 1.3 [-0.8; 3.4]  p=0.215 |
| MDT log | **0.003** | -0.1 [-0.3; 0.0]  p=0.111 | 0.001 [-0.007; 0.009]  p=0.770 | **0.002** | 0.1 [-0.1; 0.3]  p=0.504 | **0.4 [0.2; 0.6]**  **p=0.001** | **0.3 [0.0; 0.5]**  **p=0.019** |
| VDT | **0.008** | 0.3 [-0.3; 0.9]  p=0.293 | -0.011 [-0.037; 0.016]  p=0.436 | **0.009** | -0.1 [-0.9; 0.7]  p=0.839 | **-1.0 [-1.7; -0.3]**  **p=0.004** | **-1.0 [-1.8; -0.1]**  **p=0.021** |
| MPT log | **0.049** | -0.1 [-0.3; 0.1]  p=0.224 | 0.000 [-0.008; 0.008]  p=0.984 | **0.022** | **-0.3 [-0.5; -0.1]**  **p=0.010** | 0.0 [-0.2; 0.2]  p=0.890 | **0.3 [0.0; 0.5]**  **p=0.018** |
| MPS log | **0.027** | 0.0 [-0.2; 0.2]  p=0.872 | 0.005 [-0.004; 0.014]  p=0.261 | **0.003** | **0.4 [0.2; 0.7]**  **p=0.001** | 0.1 [-0.2; 0.3]  p=0.589 | **-0.4 [-0.6; -0.1]**  **p=0.007** |
| DMA log | 0.127 | 0.0 [0.0; 0.0]  p=0.107 | 0.001 [0.000; 0.002]  p=0.126 | 0.132 | 0.0 [0.0; 0.1]  p=0.082 | 0.0 [0.0; 0.0]  p=0.833 | 0.0 [-0.1; 0.0]  p=0.063 |
| WUR log | 0.209 | 0.1 [-0.1; 0.2]  p=0.328 | -0.002 [-0.007; 0.004]  p=0.509 | 0.067 | 0.1 [-0.1; 0.2]  p=0.443 | 0.2 [0.0; 0.3]  p=0.021 | 0.1 [-0.1; 0.3]  p=0.201 |
| PPT TA log | **<0.001** | -0.1 [-0.1; 0.0]  p=0.009 | 0.002 [-0.001; 0.005]  p=0.109 | **<0.001** | **-0.2 [-0.3; -0.1]**  **p<0.001** | **-0.2 [-0.2; -0.1]**  **p<0.001** | 0.0 [0.0; 0.1]  p=0.395 |
| PPT Delt log | **<0.001** | -0.2 [-0.3; -0.1]  p<0.001 | 0.003 [-0.001; 0.006]  p=0.112 | **<0.001** | **-0.3 [-0.4; -0.2]**  **p<0.001** | **-0.2 [-0.3; -0.1]**  **p<0.001** | 0.1 [0.0; 0.2]  p=0.103 |
| PPT ExtDig log | **<0.001** | -0.1 [-0.2; 0.0]  p=0.023 | 0.002 [-0.001; 0.006]  p=0.218 | **<0.001** | **-0.3 [-0.4; -0.2]**  **p<0.001** | **-0.2 [-0.3; -0.1]**  **p<0.001** | 0.1 [-0.1; 0.2]  p=0.347 |
| PPT Rectfem log | **<0.001** | -0.1 [-0.2; 0.0]  p=0.003 | 0.002 [-0.001; 0.006]  p=0.187 | **<0.001** | **-0.2 [-0.3; -0.1]**  **p<0.001** | **-0.2 [-0.3; -0.1]**  **p<0.001** | 0.0 [-0.1; 0.1]  p=0.766 |

DM1: myotonic dystrophy type 1; DM2: myotonic dystrophy type 2; CG: healthy control group; CDT: cold detection threshold; WDT: warm detection threshold; TSL: thermal sensory limen; PHS: paradoxical heat sensation; CPT: cold pain threshold; HPT: heat pain threshold; MDT: mechanical detection threshold; VDT: vibration detection threshold; MPT: mechanical pain threshold; MPS: mechanical pain sensitivity; DMA: dynamic mechanical allodynia; WUR: wind-up ratio; PPT: pressure pain threshold; TA: thenar muscle; Delt; deltoid muscle; Rectfem: rectus femoris muscle; ExtDig: extensor digitorum communis muscle; GLM: generalized linear model, F: female, M: male; β: regression coefficient; 95%-CI: 95% confidence interval
