# Supplementary material for: Characterization of the neuropathic pain component contributing to myalgia in patients with myotonic dystrophy type 1 and 2
Source: Front Neurol. 2024 Aug 13;15:1414140. doi: 10.3389/fneur.2024.1414140 (PMC11347447; doi:10.3389/fneur.2024.1414140)
Supplement: Supplementary file 2 [file Table_2.docx]

**Supplementary table 1: BMI and gender adjusted comparisons between DM2 and DM1 patients**

| **Linear GLM** | | | | |
| --- | --- | --- | --- | --- |
| **Outcome** | **Omnibus test p-value** | **Female vs  male (ref)** β [95%-CI] | **BMI** β [95%-CI] | **DM2 vs DM1 (ref)** β [95%-CI] |
| Intraepidermal nerve fiber density (IENFD) per mm^2^ | | | | |
| proximal | 0.233 | 0.60 [-1.49; 2.69]  p=0.571 | -0.15 [-0.37; 0.07]  p=0.170 | -1.4 [-3.5; 0.8] p=0.206 |
| distal | **0.031** | -0.62 [-1.98; 0.74]  p=0.375 | **-0.22 [-0.36; -0.07]**  **p=0.003** | 0.1 [-1.3; 1.5] p=0.909 |
| ratio proximal/distal | 0.442 | 0.35 [-0.67; 1.37]  p=0.500 | 0.07 [-0.03; 0.18]  p=0.178 | 0.2 [-0.8; 1.3] p=0.643 |
| Brief pain inventory (BPI) | | | | |
| Pain intensity last 24h, NRS | | | | |
| worst | 0.944 | 0.00 [-1.27; 1.26]  p=0.995 | -0.02 [-0.15; 0.11]  p=0.736 | 0.4 [-0.9; 1.7] p=0.557 |
| average | 0.611 | -0.67 [-1.71; 0.36]  p=0.201 | 0.01 [-0.10; 0.11]  p=0.873 | 0.3 [-0.8; 1.3] p=0.634 |
| mildest | 0.957 | -0.26 [-1.23; 0.71]  p=0.596 | 0.01 [-0.09; 0.10]  p=0.907 | 0.1 [-0.9; 1.1] p=0.865 |
| present | 0.106 | -0.76 [-2.14; 0.61]  p=0.276 | 0.06 [-0.08; 0.20]  p=0.388 | 1.4 [0.0; 2.8] p=0.054 |
| Pain severity | 0.599 | -0.43 [-1.42; 0.57]  p=0.401 | 0.01 [-0.09; 0.12]  p=0.794 | 0.5 [-0.5; 1.6] p=0.309 |
| Pain interference | **0.026** | -0.71 [-1.78; 0.36]  p=0.192 | 0.07 [-0.04; 0.18]  p=0.242 | **1.3 [0.2; 2.4] p=0.017** |
| Pain DETECT | | | | |
| Neuropathic pain quality sum score | 0.154 | -1.39 [-5.13; 2.35]  p=0.467 | 0.34 [-0.04; 0.72]  p=0.080 | 1.9 [-1.9; 5.8] p=0.322 |
| Final score | 0.142 | -1.41 [-5.30; 2.47]  p=0.475 | 0.34 [-0.06; 0.74]  p=0.094 | 2.4 [-1.6; 6.4] p=0.246 |
| Pain disability index (PDI) | 0.110 | -0.41 [-8.64; 7.82]  p=0.922 | 0.40 [-0.44; 1.24]  p=0.350 | 8.8 [0.3; 17.2] p=0.043 |
| **Ordinal logistic GLM** | | | | |
| **Outcome** | **Omnibus test p-value** | **F vs M(ref)** OR [95%-CI]  p-value | **Age** OR [95%-CI] p-value | **DM2 vs DM1 (ref)** OR [95%-CI] p-value |
| Pain DETECT | | | | |
| Pain pattern | 0.050 | 0.4 [0.1; 1.2]  p=0.089 | 1. [0.9; 1.1] 2. p=0.675 | **0.3 [0.1; 0.9]**  **p=0.040** |
| Neuropathic pain category | 0.271 | 0.8 [0.3; 2.4]  p=0.688 | 1.1 [0.9; 1.2]  p=0.312 | 2.1 [0.6; 7.0]  p=0.217 |
| **Binomial logistic GLM** |  |  |  |  |
| **Outcome** | **Omnibus test p-value** | **F vs M(ref)** OR [95%-CI]  p-value | **Age** OR [95%-CI] p-value | **DM2 vs DM1 (ref)** OR [95%-CI] p-value |
| Current pain medication | 0.398 | 1.3 [0.4; 4.8]  p=0.696 | 1.0 [0.9; 1.1]  p=0.948 | 3.2 [0.7; 14.0]  p=0.120 |
| Neurological examination | | | | |
| DTR reduced/absent | **<0.001** | 0.5 [0.1; 1.9]  p=0.280 | 1.1 [1.0; 1.3]  p=0.109 | **0.1 [0.0; 0.3]**  **p<0.001** |
| Clinical myotoina | **<0.001** | 0.3 [0.1; 1.7]  p=0.191 | 0.9 [0.7; 1.0]  p=0.109 | **0.0 [0.0; 0.2]**  **p<0.001** |
| Brief pain inventory (BPI) |  |  |  |  |
| Pain today | 0.053 | 4.5 [1.2; 17.6]  p=0.030 | 1.0 [0.9; 1.1]  p=0.882 | 3.2 [0.8; 12.8]  p=0.099 |
| Pain DETECT – radiating pain | **0.001** | 2.5 [0.6; 10.3]  p=0.191 | 1.0 [0.9; 1.2]  p=0.660 | **11.6 [2.8; 48.0]**  **p=0.001** |

DM1: myotonic dystrophy type 1; DM2: myotonic dystrophy type 2; BMI: body mass index; NRS: numeric rating scale; DTR: deep tendon reflex; GLM: generalized linear model; β: regression coefficient; OR: odds ratio; 95%-CI: 95% confidence interval

| **Outcome** | **Omnibus test GLM p-value** | **F vs M(ref)** β [95%-CI]  p-value | **BMI** β [95%-CI] p-value | **Overall group effect p-value** | **DM1 vs CG (ref)** β [95%-CI] p-value | **DM2 vs CG (ref)**  β [95%-CI] p-value | **DM2 vs DM1 (ref)** β [95%-CI] p-value |
| --- | --- | --- | --- | --- | --- | --- | --- |
| QST at the thigh and PPT at indicated measure sites | | | | | | | |
| CDT log | **<0.001** | -0.03 [-0.12; 0.06]  p=0.507 | **0.02 [0.01; 0.03]**  **p<0.001** | **<0.001** | **0.1 [0.0; 0.3]**  **p=0.008** | **0.3 [0.2; 0.4]**  **p<0.001** | **0.1 [0.0; 0.2]**  **p=0.011** |
| WDT log | **<0.001** | -0.07 [-0.15; 0.02]  p=0.119 | **0.01 [0.00; 0.02]**  **p=0.027** | **<0.001** | 0.0 [-0.1; 0.1]  p=0.602 | **0.2 [0.1; 0.3]**  **p<0.001** | **0.2 [0.1; 0.3]**  **p=0.001** |
| TSL log | **<0.001** | -0.06 [-0.15; 0.02]  p=0.161 | **0.01 [0.00; 0.02]**  **p=0.004** | **<0.001** | 0.1 [0.0; 0.2]  p=0.073 | **0.2 [0.2; 0.3]**  **p<0.001** | **0.2 [0.0; 0.3]**  **p=0.005** |
| CPT | 0.299 | -2.83 [-7.49; 1.82]  p=0.233 | 0.27 [-0.26; 0.79]  p=0.322 | 0.388 | 4.0 [-1.7; 9.7]  p=0.171 | 1.4 [-3.9; 6.7]  p=0.606 | -2.6 [-8.3; 3.1]  p=0.369 |
| HPT | 0.067 | -0.40 [-2.05; 1.25]  p=0.636 | 0.14 [-0.05; 0.33]  p=0.142 | 0.084 | -1.8 [-3.9; 0.2]  p=0.075 | 0.3 [-1.6; 2.2]  p=0.737 | **2.2 [0.2; 4.2]**  **p=0.035** |
| MDT log | **0.001** | -0.11 [-0.29; 0.06]  p=0.210 | 0.02 [0.00; 0.04]  p=0.096 | **0.004** | 0.1 [-0.1; 0.3]  p=0.522 | **0.3 [0.1; 0.5]**  **p=0.002** | **0.3 [0.0; 0.5]**  **p=0.021** |
| VDT | **0.004** | 0.24 [-0.38; 0.85]  p=0.452 | -0.05 [-0.12; 0.02]  p=0.152 | **0.008** | 0.0 [-0.8; 0.7]  p=0.966 | **-1.0 [-1.7; -0.3]**  **p=0.006** | **-1.0 [-1.7; -0.2]**  **p=0.011** |
| MPT log | 0.050 | -0.11 [-0.29; 0.07]  p=0.240 | 0.00 [-0.02; 0.02]  p=0.930 | 0.015 | **-0.3 [-0.5; -0.1]**  **p=0.008** | 0.0 [-0.2; 0.2]  p=0.870 | **0.3 [0.1; 0.5]**  **p=0.012** |
| MPS log | **0.022** | 0.01 [-0.20; 0.22]  p=0.916 | 0.02 [-0.01; 0.04]  p=0.186 | **0.004** | **0.4 [0.2; 0.7]**  **p=0.002** | 0.1 [-0.2; 0.3]  p=0.619 | **-0.3 [-0.6; -0.1]**  **p=0.007** |
| DMA log | 0.264 | -0.02 [-0.04; 0.01]  p=0.149 | 0.00 [0.00; 0.00]  p=0.541 | 0.271 | 0.0 [0.0; 0.1]  p=0.154 | 0.0 [0.0; 0.0]  p=0.995 | 0.0 [-0.1; 0.0]  p=0.152 |
| WUR log | 0.235 | 0.06 [-0.07; 0.18]  p=0.373 | 0.00 [-0.02; 0.01]  p=0.742 | 0.082 | 0.1 [-0.1; 0.2]  p=0.353 | 0.2 [0.0; 0.3]  p=0.026 | 0.1 [-0.1; 0.2]  p=0.252 |
| PPT TA log | **<0.001** | -**0.07 [-0.14; -0.01]**  **p=0.026** | 0.01 [0.00; 0.01]  p=0.101 | **<0.001** | **-0.2 [-0.3; -0.1]**  **p<0.001** | **-0.2 [-0.2; -0.1]**  **p<0.001** | 0.1 [0.0; 0.1]  p=0.209 |
| PPT Delt log | **<0.001** | **-0.17 [-0.26; -0.09]**  **p<0.001** | 0.00 [-0.01; 0.01]  p=0.954 | **<0.001** | **-0.3 [-0.4; -0.2]**  **p<0.001** | **-0.2 [-0.3; -0.1]**  **p=0.001** | **0.1 [0.0; 0.2]**  **p=0.016** |
| PPT ExtDig log | **<0.001** | **-0.09 [-0.17; 0.00]**  **p=0.041** | 0.00 [-0.01; 0.01]  p=0.370 | **<0.001** | **-0.3 [-0.4; -0.2]**  **p<0.001** | **-0.2 [-0.3; -0.1]**  **p<0.001** | 0.1 [0.0; 0.2]  p=0.178 |
| PPT Rectfem log | **<0.001** | **-0.11 [-0.19; -0.03]**  **p=0.007** | 0.00 [0.00; 0.01]  p=0.307 | **<0.001** | **-0.2 [-0.3; -0.1]**  **p<0.001** | **-0.2 [-0.3; -0.1]**  **p<0.001** | 0.0 [-0.1; 0.1]  p=0.502 |

DM1: myotonic dystrophy type 1; DM2: myotonic dystrophy type 2; CG: healthy control group; BMI: body mass index; CDT: cold detection threshold; WDT: warm detection threshold; TSL: thermal sensory limen; PHS: paradoxical heat sensation; CPT: cold pain threshold; HPT: heat pain threshold; MDT: mechanical detection threshold; VDT: vibration detection threshold; MPT: mechanical pain threshold; MPS: mechanical pain sensitivity; DMA: dynamic mechanical allodynia; WUR: wind-up ratio; PPT: pressure pain threshold; TA: thenar muscle; Delt; deltoid muscle; Rectfem: rectus femoris muscle; ExtDig: extensor digitorum communis muscle; GLM: generalized linear model, F: female, M: male; β: regression coefficient; 95%-CI: 95% confidence interval

|  | **Omnibus test GLM p-value** | **BMI** β [95%-CI] p-value | **DM2 vs DM1 (ref)** β [95%-CI] p-value |
| --- | --- | --- | --- |
| QST at the dorsum of the hand | | | |
| z-score CDT | 0.134 | -0.01 [-0.06; 0.04]  p=0.689 | -0.4 [-0.9; 0.0]  p=0.066 |
| z-score WDT | **0.001** | **-0.08 [-0.16; -0.01]**  **p=0.032** | **-1.0 [-1.7; -0.2]**  **p=0.010** |
| z-score TSL | **0.001** | **-0.07 [-0.12; -0.01]**  **p=0.016** | **-0.7 [-1.3; -0.1]**  **p=0.015** |
| z-score CPT | 0.168 | 0.06 [0.00; 0.13]  p=0.060 | -0.3 [-0.9; 0.4]  p=0.392 |
| z-score HPT | 0.071 | -0.04 [-0.11; 0.03]  p=0.254 | -0.6 [-1.3; 0.1]  p=0.087 |
| z-score MDT | 0.706 | -0.01 [-0.08; 0.06]  p=0.716 | -0.2 [-0.9; 0.5]  p=0.523 |
| z-score VDT | 0.213 | -0.02 [-0.09; 0.04]  p=0.479 | -0.5 [-1.1; 0.2]  p=0.158 |
| z-score MPT | **0.017** | 0.00 [-0.07; 0.06]  p=0.910 | **-1.0 [-1.6; -0.3]**  **p=0.005** |
| z-score MPS | **0.010** | 0.02 [-0.04; 0.08]  p=0.528 | **-1.0 [-1.6; -0.4]**  **p=0.002** |
| z-score DMA | 0.802 | 0.10 [-0.21; 0.40]  p=0.540 | 0.1 [-0.4; 0.7]  p=0.677 |
| z-score WUR | 0.553 | 0.02 [-0.03; 0.08]  p=0.381 | -0.6 [-3.7; 2.4]  p=0.687 |
| z-score PPT_TA | 0.575 | -0.04 [-0.12; 0.04]  p=0.298 | 0.2 [-0.6; 0.9]  p=0.653 |

DM1: myotonic dystrophy type 1; DM2: myotonic dystrophy type 2; CG: healthy control group; BMI: body mass index; CDT: cold detection threshold; WDT: warm detection threshold; TSL: thermal sensory limen; PHS: paradoxical heat sensation; CPT: cold pain threshold; HPT: heat pain threshold; MDT: mechanical detection threshold; VDT: vibration detection threshold; MPT: mechanical pain threshold; MPS: mechanical pain sensitivity; DMA: dynamic mechanical allodynia; WUR: wind-up ratio; PPT: pressure pain threshold; TA: thenar muscle; GLM: generalized linear model, β: regression coefficient; 95%-CI: 95% confidence interval
