# Supplementary material for: Characterization of the neuropathic pain component contributing to myalgia in patients with myotonic dystrophy type 1 and 2
Source: Front Neurol. 2024 Aug 13;15:1414140. doi: 10.3389/fneur.2024.1414140 (PMC11347447; doi:10.3389/fneur.2024.1414140)
Supplement: Supplementary file 3 [file Table_3.docx]

**Supplementary table3: Nerve conduction studies**

|  |  | **Sensory Nerves** | | | | **Motor Nerves** | | | | | |
| --- | --- | --- | --- | --- | --- | --- | --- | --- | --- | --- | --- |
|  |  | **Radial Nerve** | | **Sural Nerve** | | **Peroneal Nerve** | | | **Ulnar Nerve** | | |
| **Pt** | **Diagnosis** | **Amplitude (µV)** | **CV (m/s)** | **Amplitude (µV)** | **CV (m/s)** | **Dml (ms)** | **Amplitude (mV)** | **CV (m/s)** | **Dml (ms)** | **Amplitude (mV)** | **CV (m/s)** |
| 1 | DM1 | 18.0 | 77.0 | 10.0 | 40.0 | 4.1 | 3.5 | 40.3 | 2.5 | 8.3 | 50.0 |
| 2 | DM1 | 66.0 | 64.3 | 38.0 | 49.2 | 3.9 | 8.2 | 52.8 | 3.0 | 14.6 | 63.8 |
| 4 | DM1 | 36.6 | 61.1 | 25.6 | 44.2 | 3.2 | 6.6 | 52.0 | 2.7 | 10.7 | 52.3 |
| 5 | DM1 | 36.6 | 68.3 | 34.9 | 62.5 | 3.5 | 7.4 | 49.7 | 2.3 | 6.0 | 59.2 |
| 10 | DM1 | 39.7 | 56.1 | 35.5 | 45.9 | 3.5 | 11.1 | 51.2 | 3.2 | 16.3 | 54.2 |
| 12 | DM1 | 75.1 | 72.4 | 42.5 | 48.5 | 3.8 | 13.1 | 45.8 | 2.4 | 12.4 | 60.4 |
| 16 | DM1 | 35.8 | 60.8 | 8.6 | 42.6 | 4.0 | 7.0 | 45.6 | 3.2 | 12.4 | 51.2 |
| 17 | DM1 | 69.4 | 62.9 | 20.4 | 48.1 | 3.7 | 7.6 | 52.2 | 2.8 | 14.2 | 55.1 |
| 19 | DM1 | 25.1 | 66.8 | 22.7 | 41.7 | 3.9 | 9.8 | 47.0 | 3.0 | 10.0 | 56.5 |
| 21 | DM1 | 23.7 | 58.3 | 10.4 | 45.5 | 3.6 | 7.6 | 42.9 | 3.1 | 12.1 | 51.3 |
| 23 | DM1 | 25.9 | 65.6 | 18.7 | 51.8 | 4.3 | 4.7 | 45.5 | 3.1 | 9.7 | 58.2 |
| 27 | DM1 | 33.2 | 65.8 | 30.9 | 56.3 | 4.4 | 9.8 | 48.6 | 2.6 | 11.7 | 55.6 |
| 29 | DM1 | 35.0 | 68.5 | 22.6 | 56.3 | 3.4 | 12.0 | 53.5 | 2.7 | 16.3 | 65.0 |
| 33 | DM1 | 30.4 | 57.9 | 20.1 | 38.9 | 5.7 | 2.7 | 45.8 | 4.0 | 17.1 | 57.8 |
| 37 | DM1 | 53.0 | 54.5 | 33.8 | 47.8 | 3.9 | 13.1 | 59.0 | 3.8 | 14.0 | 55.0 |
| 38 | DM1 | 44.9 | 53.9 | 9.1 | 70.9 | 4.9 | 18.3 | 62.8 | 2.5 | 13.7 | 52.8 |
| 42 | DM1 | 21.6 | 54.1 | 7.1 | 44.6 | 4.3 | 13.2 | 47.1 | 2.9 | 16.6 | 58.7 |
| 50 | DM1 | 50.6 | 51.0 | 21.7 | 41.5 | 4.4 | 6.4 | 42.3 | 2.9 | 10.7 | 53.6 |
| 51 | DM1 | 46.8 | 62.5 | 9.6 | 45.2 | 4.9 | 3.7 | 42.3 | 2.5 | 8.0 | 54.6 |
| 52 | DM1 | 45.5 | 70.5 | 14.5 | 43.7 | 3.7 | 2.0 | 49.2 | 2.6 | 6.4 | 57.5 |
| 53 | DM1 | 40.1 | 65.8 | 19.4 | 59.2 | 3.7 | 9.2 | 50.2 | 2.3 | 14.0 | 59.1 |
| 3 | DM2 | 25.4 | 59.2 | 8.2 | 39.4 | 3.6 | 9.5 | 46.4 | 2.9 | 11.3 | 53.0 |
| 6 | DM2 | 36.4 | 61.2 | 18.3 | 51.4 | 5.1 | 3.7 | 47.2 | 3.0 | 12.5 | 61.3 |
| 7 | DM2 | 31.1 | 69.1 | 19.9 | 42.9 | 4.4 | 6.2 | 44.6 | 2.7 | 12.6 | 56.3 |
| 8 | DM2 | 18.8 | 61.9 | 21.3 | 45.2 | 3.9 | 4.8 | 47.3 | 3.0 | 19.2 | 52.0 |
| 9 | DM2 | 34.9 | 67.6 | 10.4 | 53.2 | 4.1 | 4.7 | 50.9 | 3.1 | 9.0 | 62.9 |
| 11 | DM2 | 29.6 | 69.1 | 18.3 | 55.2 | 4.1 | 5.7 | 43.9 | 1.9 | 12.0 | 49.1 |
| 13 | DM2 | 40.0 | 60.8 | 13.4 | 42.6 | 3.5 | 17.9 | 55.8 | 4.5 | 5.3 | 48.9 |
| 14 | DM2 | 25.3 | 61.9 | 19.8 | 45.0 | 4.0 | 5.1 | 43.7 | 2.9 | 6.2 | 55.1 |
| 15 | DM2 | 24.9 | 69.4 | 13.5 | 54.6 | 4.0 | 5.0 | 42.7 | 2.7 | 12.5 | 57.4 |
| 18 | DM2 | 25.4 | 63.6 | 10.8 | 52.1 | 4.2 | 9.2 | 41.6 | 3.5 | 15.6 | 54.4 |
| 20 | DM2 | 31.5 | 61.2 | 14.5 | 41.5 | 5.6 | 10.7 | 46.8 | 3.5 | 16.2 | 53.8 |
| 22 | DM2 | 49.0 | 70.4 | 19.4 | 52.6 | 3.8 | 5.6 | 47.9 | 2.1 | 15.0 | 63.2 |
| 24 | DM2 | 24.1 | 77.5 | 6.5 | 46.6 | 4.3 | 6.8 | 44.9 | 2.6 | 15.0 | 59.8 |
| 25 | DM2 | 24.5 | 61.2 | 7.9 | 54.3 | 4.0 | 7.4 | 53.1 | 2.6 | 17.1 | 56.1 |
| 26 | DM2 | 42.8 | 63.3 | 33.1 | 49.0 | 5.2 | 5.0 | 48.1 | 2.4 | 14.5 | 62.8 |
| 28 | DM2 | 26.1 | 55.6 | 5.1 | 42.2 | 4.3 | 2.7 | 42.9 | 3.4 | 9.6 | 52.7 |
| 30 | DM2 | 40.8 | 64.2 | 10.3 | 46.2 | 3.9 | 8.1 | 46.8 | 2.5 | 17.2 | 64.0 |
| 31 | DM2 | 22.4 | 54.7 | 19.2 | 44.6 | 5.9 | 4.1 | -99.0 | 3.5 | 13.6 | 57.1 |
| 32 | DM2 | 44.0 | 69.4 | 22.4 | 50.8 | 4.4 | 9.9 | 48.4 | 2.2 | 15.3 | 59.0 |
| 34 | DM2 | 27.4 | 61.5 | 34.2 | 47.3 | 5.3 | 6.3 | 42.9 | 3.2 | 15.1 | 55.4 |
| 35 | DM2 | 28.7 | 76.3 | 10.3 | 51.5 | 3.3 | 7.1 | 49.8 | 2.2 | 11.7 | 61.6 |
| 36 | DM2 | 36.7 | 52.9 | 19.3 | 51.9 | 3.8 | 9.8 | 44.6 | 3.0 | 16.8 | 54.9 |
| 39 | DM2 | 24.4 | 67.2 | 18.8 | -99.0 | 3.9 | 6.4 | 45.2 | 2.7 | 12.5 | 61.3 |
| 40 | DM2 | 38.0 | 65.0 | 34.5 | 61.5 | 3.6 | 6.2 | 48.7 | 2.7 | 9.5 | 59.2 |
| 41 | DM2 | 13.6 | 56.5 | 7.1 | 45.1 | 4.4 | 8.5 | 43.1 | 3.2 | 11.0 | 51.6 |
| 43 | DM2 | 38.4 | 59.9 | 13.2 | 50.3 | 4.7 | 9.4 | 47.0 | 3.3 | 11.4 | 61.7 |
| 44 | DM2 | 29.6 | 60.0 | 6.8 | 36.7 | 3.8 | 4.8 | 42.5 | 3.2 | 9.0 | 57.0 |
| 45 | DM2 | 26.7 | 61.5 | 24.6 | 44.1 | 4.0 | 10.1 | 46.0 | 2.2 | 17.8 | 54.3 |
| 46 | DM2 | 28.9 | 62.9 | 12.7 | 43.0 | 3.9 | 12.2 | 47.9 | 3.1 | 18.9 | 60.5 |
| 47 | DM2 | 36.5 | 55.9 | 12.7 | 40.4 | 3.5 | 3.7 | 43.0 | 2.7 | 12.8 | 54.9 |
| 48 | DM2 | 25.6 | 62.8 | 27.1 | 34.6 | 4.2 | 5.0 | 48.6 | 2.7 | 20.2 | 62.1 |
| 49 | DM2 | 29.9 | 67.9 | 16.0 | 50.8 | 3.9 | 7.3 | 42.8 | 3.3 | 13.4 | 58.4 |
| 54 | CG | 42.7 | 65.8 | 21.7 | 43.3 | 4.1 | 6.4 | 47.4 | 2.3 | 18.1 | 59.7 |
| 55 | CG | 39.6 | 70.0 | 29.1 | 47.9 | 3.9 | 8.3 | 45.8 | 2.4 | 14.1 | -99.0 |
| 56 | CG | 14.6 | 55.0 | 16.2 | 39.0 | 3.5 | 9.4 | 43.1 | 2.7 | 11.1 | 58.3 |
| 58 | CG | 38.3 | 68.5 | 19.3 | 61.6 | 3.2 | 18.6 | 53.9 | 2.2 | 20.5 | 56.0 |
| 59 | CG | 20.1 | 57.9 | 8.5 | 42.6 | 4.1 | **1.2** | 32.3 | 3.4 | 12.3 | 57.1 |
| 60 | CG | 31.6 | 63.3 | 20.8 | 48.1 | 4.3 | 6.7 | 45.4 | 2.9 | 15.1 | 61.2 |
| 61 | CG | 14.5 | 65.9 | 7.5 | 44.4 | 4.2 | 5.0 | 39.7 | 2.3 | 14.2 | 54.7 |
| 62 | CG | 45.6 | 70.4 | 34.1 | 49.3 | 3.9 | 11.8 | 51.7 | 2.2 | 13.0 | 63.0 |
| 63 | CG | 14.8 | 55.3 | 10.9 | 50.3 | 3.9 | 2.5 | 42.0 | 2.5 | 14.5 | 58.9 |
| 64 | CG | 32.6 | 69.1 | 20.9 | 52.4 | 3.8 | 10.3 | 51.5 | 2.9 | 17.3 | 67.3 |
| 65 | CG | 54.8 | 70.5 | 26.4 | 48.5 | 3.9 | 3.9 | 50.4 | 2.5 | 19.8 | 66.4 |
| 66 | CG | 20.0 | 68.4 | 12.6 | 40.0 | 4.8 | 7.7 | 48.0 | 3.1 | 15.1 | 57.8 |
| 67 | CG | 47.4 | 72.2 | 31.4 | 46.1 | 3.9 | 12.5 | 53.4 | 2.3 | 20.2 | 68.5 |
| 68 | CG | 51.1 | 65.9 | 20.2 | 51.4 | 3.7 | 5.1 | 50.4 | 2.7 | 12.8 | 69.1 |
| 70 | CG | 60.4 | 66.0 | 30.8 | 52.6 | 4.8 | **2.0** | 49.8 | 2.5 | 17.9 | 63.4 |
| 71 | CG | 35.3 | 63.4 | 15.8 | 49.6 | 3.1 | 10.3 | 51.9 | 2.4 | 17.7 | 59.7 |
| 72 | CG | 62.0 | 68.5 | 15.6 | 51.3 | 3.6 | 9.2 | 51.2 | 3.0 | 9.5 | 48.7 |
| 73 | CG | 27.9 | 63.6 | 18.0 | 50.0 | 4.2 | 9.2 | 52.0 | 2.3 | 16.9 | 67.0 |
| 74 | CG | 52.8 | 62.2 | 16.7 | 43.0 | 4.4 | 10.6 | 56.6 | 3.3 | 12.9 | 58.3 |
| 75 | CG | 32.3 | 60.8 | 11.4 | 53.3 | 3.9 | 8.8 | 51.5 | 3.0 | 15.3 | 61.2 |
| 76 | CG | 41.8 | 68.8 | 19.4 | 52.9 | 3.6 | 8.6 | 56.0 | 2.3 | 13.5 | 68.1 |
| 77 | CG | 39.6 | 70.4 | 22.9 | 44.1 | 3.2 | 13.4 | 50.8 | 2.5 | 16.0 | 66.6 |
| 78 | CG | 18.8 | 51.1 | 12.1 | 38.3 | 4.9 | 4.6 | 46.0 | 3.7 | 11.0 | 59.1 |
| 81 | CG | 16.7 | 61.7 | 6.3 | 44.1 | 4.5 | 5.4 | 43.6 | 2.6 | 10.9 | 61.0 |
| 83 | CG | 43.0 | 60.0 | 18.0 | 43.0 | 4.4 | 4.5 | 48.0 | 3.2 | 19.0 | 60.0 |

DM1: myotonic dystrophy type 1; DM2: myotonic dystrophy type 2; CG: control group; Dml: distal motor latency; m/s: meters per second; CV: conduction velocity; µV: micro Volt; Pt: patient. Text in bold: pathological values.
